# Fetal manipulation of maternal metabolism is a critical function of the imprinted *Igf2* gene

**DOI:** 10.1016/j.cmet.2023.06.007

**Published:** 2023-07-11

**Authors:** Jorge Lopez-Tello, Hannah E.J. Yong, Ionel Sandovici, Georgina K.C. Dowsett, Efthimia R. Christoforou, Esteban Salazar-Petres, Rebecca Boyland, Tina Napso, Giles S.H. Yeo, Brian Y.H. Lam, Miguel Constancia, Amanda N. Sferruzzi-Perri

**Affiliations:** 1 Centre for Trophoblast Research, Department of Physiology, Development and Neuroscience, University of Cambridge, Cambridge CB2 3EG, UK; 2 Singapore Institute for Clinical Sciences (SICS), Agency for Science, Technology and Research (A*STAR), 30 Medical Drive, Singapore 117609, Singapore; 3 Department of Obstetrics and Gynaecology and National Institute for Health Research Cambridge Biomedical Research Centre, Cambridge CB2 0SW, UK; 4 Medical Research Council (MRC) Metabolic Diseases Unit, Wellcome-MRC Institute of Metabolic Science and, University of Cambridge, Cambridge CB2 0QQ, UK; 5 Royal Devon and Exeter Hospital NHS Trust, Barrack Rd, Exeter EX2 5DW, UK

## Abstract

Maternal-offspring interactions in mammals involve both cooperation and conflict. The fetus has evolved ways to manipulate maternal physiology to enhance placental nutrient transfer, but the mechanisms involved remain unclear. The imprinted *Igf2* gene is highly expressed in murine placental endocrine cells. Here, we show that *Igf2* deletion in these cells impairs placental endocrine signaling to the mother, without affecting placental morphology. *Igf2* controls placental hormone production, including prolactins, and is crucial to establish pregnancy-related insulin resistance and to partition nutrients to the fetus. Consequently, fetuses lacking placental endocrine *Igf2* are growth restricted and hypoglycemic. Mechanistically, *Igf2* controls protein synthesis and cellular energy homeostasis, actions dependent on the placental endocrine cell type. *Igf2* loss also has additional long-lasting effects on offspring metabolism in adulthood. Our study provides compelling evidence for an intrinsic fetal manipulation system operating in placenta that modifies maternal metabolism and fetal resource allocation, with long-term consequences for offspring metabolic health.

## Graphical abstract

**Graphical abstract:**
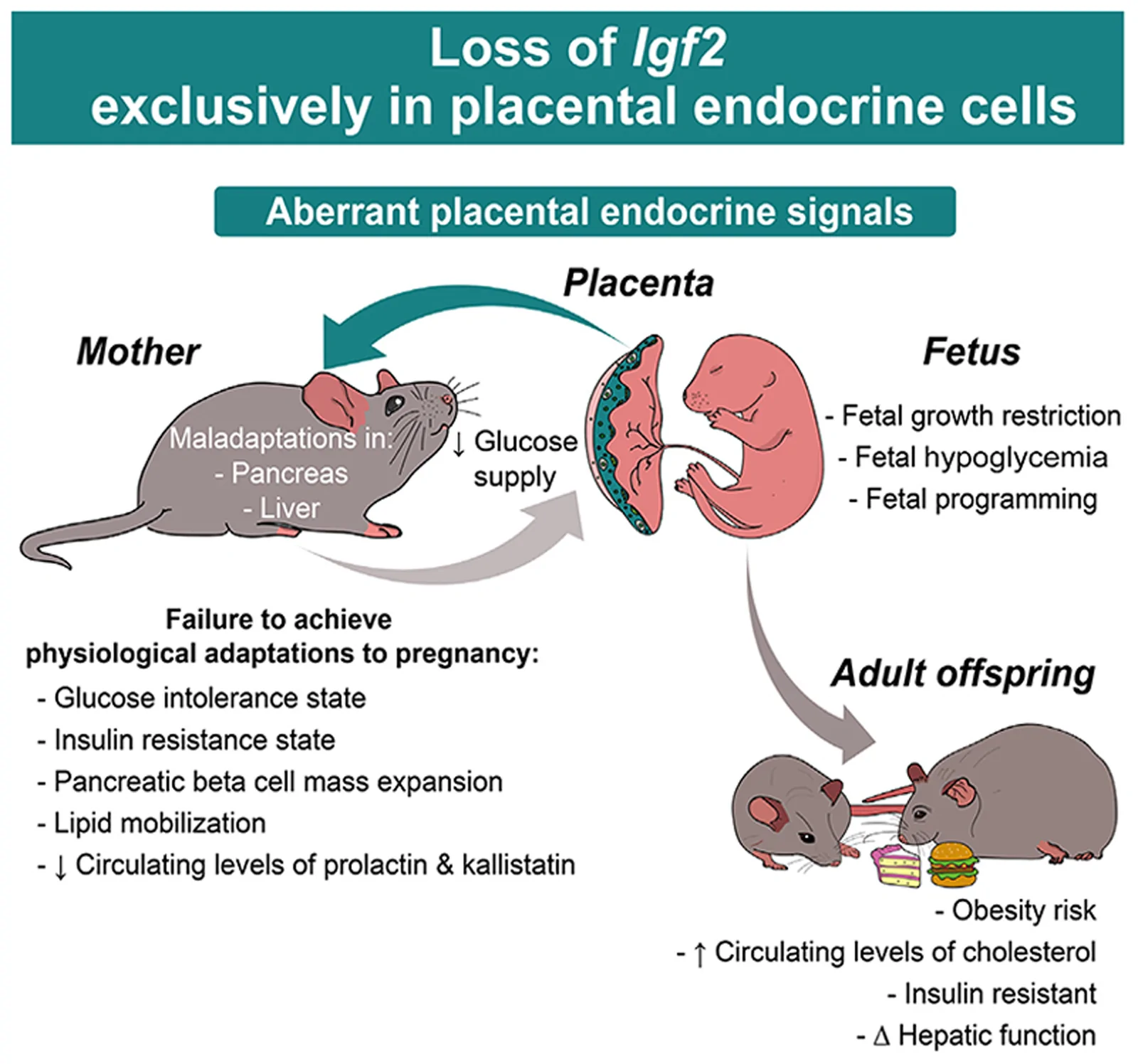

## Introduction

Maternal-offspring interactions are largely viewed as cooperative, as both the mother and fetus have an interest in each other’s wellbeing and survival. Pregnancy enhances metabolic demands on the mother to increase nutrient availability for fetal growth and development.^1,2^ However, the fetus will seek to extract more resources than the mother is prepared to give because parents and offspring do not share all genes and future maternal reproduction can be negatively affected if too much investment is placed in the current offspring.^3^ In the context of pregnancy, this genetically-driven “conflict” over maternal resources was evolutionary selected concomitantly with the appearance of first placental mammals around 150 million years ago.^4–6^ Genomic imprinting, which co-evolved with placentation, and placental hormone production represent two important mechanisms thought to mediate fetal “manipulation” of maternal physiology for the benefit of the fetus.^7,8^

Most studies to date have shown key roles for imprinted genes in regulating the placental supply of nutrients to the fetus or in the regulation of fetal resource acquisition through actions in the mother, where these genes are also expressed.^9,10^ However, direct evidence for a role of placental imprinting or hormones as fetal manipulation systems of maternal resources is currently lacking.

## Results

### Loss of placental endocrine cell *Igf2* leads to fetal hypoglycemia and growth restriction

**Figure 1:**
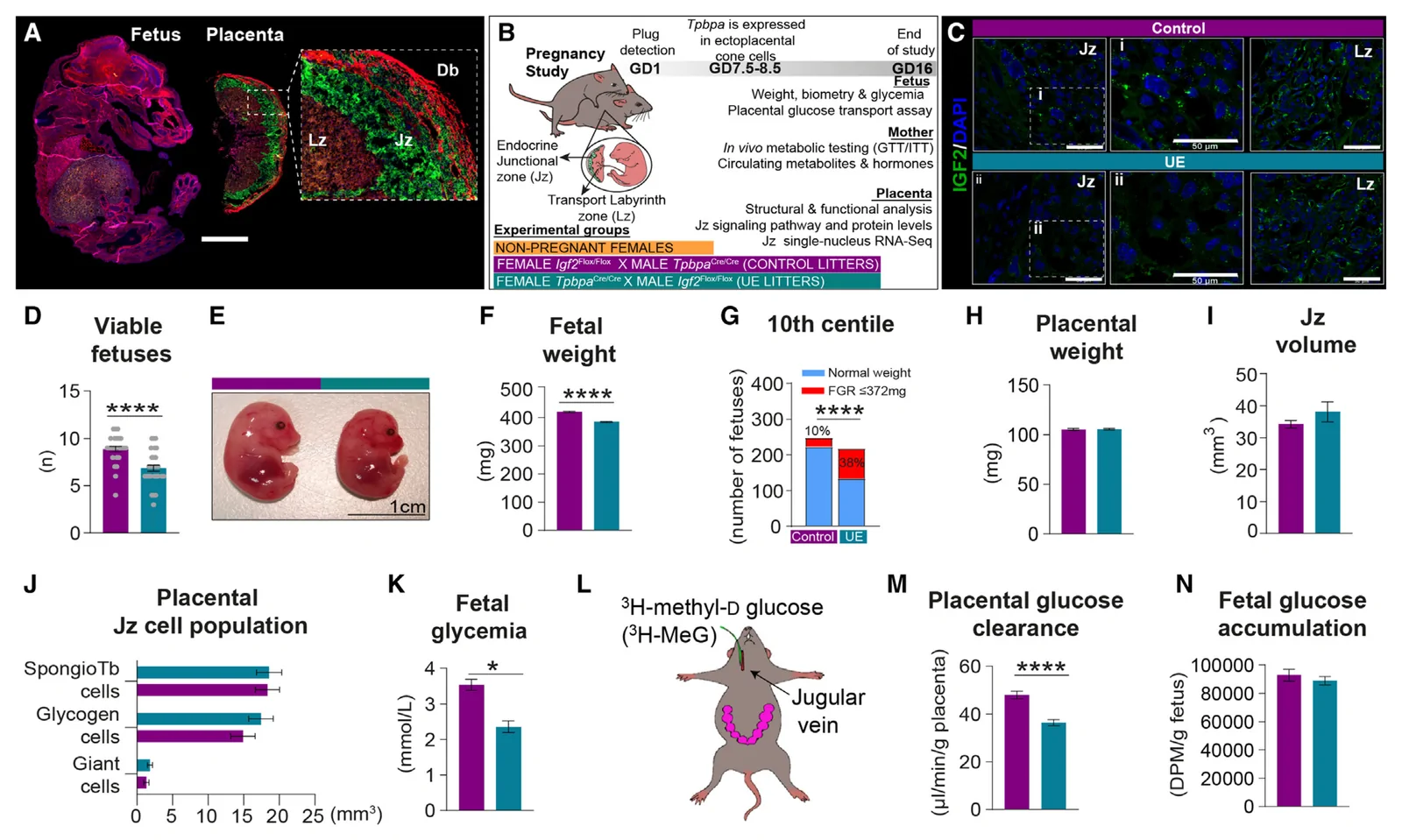
Loss of *Igf2* in the placental endocrine cells is associated with poor fetal outcomes (A) mT/mG-Cre fluorescent reporter showing that Cre is exclusively active in the placental endocrine zone (junctional zone [Jz]) and not in the transport region (labyrinth zone [Lz]), maternal decidua (Db), or the fetus. Samples collected on day 16 of gestation. Scale bar represents 2,000 µm. (B) Schematic of experimental design and mating strategy to disrupt *Igf2* in the placental Jz. (C) Representative IGF2 protein staining in the Jz and not Lz for each experimental group. Scale bars represent 50 µm. (D) Number of viable fetuses per litter by genotype (n = 28–32 litters/group). (E and F) Representative image of a fetus from each experimental group and absolute fetal weight (n = 28–32 litters/group). (G) Proportion of fetuses considered as fetal growth restricted using the 10th centile cut-off (defined as fetal weight below the 10th centile—372 mg—of the control group, n = 28–32 litters/group). (H and I) Placental weight (n = 28–32 litters/group) and Jz volume as determined by placental stereological analysis (n = 5/group). (J) Proportions of spongiotrophoblast, glycogen, and giant cell populations within the Jz (n = 4–5/group). (K) Fetal blood glucose concentration (assessed in two randomly selected fetuses per litter, n = 8–9 litters/ group). (L) Schematic of jugular vein injection with radiolabeled glucose for *in vivo* placental transport assay. (M) *In vivo* placental transport of ^3^H-methyl-D glucose (MeG) relative to placental weight (n = 7–8 litters/group). (N) Fetal glucose accumulation following injection of dam with radio-labeled MeG (n = 7–8 litters/group). Data are presented as mean ± SEM with or without individual data points shown. Viability of fetuses was analyzed with a Mann-Whitney test. Feto-placental data were analyzed by repeated measures ANOVA (MIXED model), considering the mother as the subject and the genotype as the main factor, with litter size as a covariate. *p < 0.05;****p < 0.0001. See also Data S1.

Insulin-like growth factor 2 (*Igf2*) is a paternally expressed imprinted gene, with the maternal gene copy silenced by epigenetic mechanisms.^11^ Human placental *IGF2* expression is linked with altered fetal growth in pregnancy complications, such as gestational diabetes,^12^ which may be influenced by feto-placental *IGF2* variants as suggested in two studies highlighting a significant relationship of maternal glycemia with paternally transmitted fetal *IGF2* variants.^13,14^ To better understand how placental *Igf2*, and specifically *Igf2* from endocrine cells of the placenta, may regulate maternal resource allocation, we employed a mouse model based on the Cre-*LoxP* technology, in which expression of Cre recombinase is driven by the *Tpbpa* promoter.^15^ The *Tpbpa* gene is exclusively expressed by ectoplacental cone cells that subsequently differentiate into placental endocrine cells, which form the junctional zone (Jz) layer of the mouse placenta.^15,16^ Consistent with this, *Tpbpa*^*Cre*^ is selectively active in the placental Jz of the developing conceptus (Figures 1A and S1A). *Tpbpa* is highly expressed by the Jz, with undetectable levels in the placental labyrinth zone (Lz) and the fetus (Figure 1A), as well as in different tissues during adult life (Figures S1B and S1C). *Igf2* is widely expressed by the developing mouse conceptus,^17^ including in the placental Jz, with varying levels detectable in adult tissues (Figure S1D).

We generated reciprocal crosses between homozygous *Tpbpa*^*Cre*^ and homozygous *Igf2*^*Flox*^ mice and obtained dams that carried offspring with either a deletion of the paternally inherited *Igf2* allele in Jz (with diminished *Igf2* production from the endocrine cells in whole litters, referred to as under-expression [UE] litters), or deletion of the maternally inherited and silent *Igf2* allele (referred to as control litters, with normal *Igf2* levels in whole litters) (Figure 1B). This experimental design that used entirely manipulated litters instead of litters with mixed genotypes, with assessments performed on day 16 of gestation (80% of the total mouse pregnancy), aimed to fully uncover the effects of UE on the maternal phenotype without the potential dilution of effects caused by the presence of control littermates. In litters of mixed genotypes, we have previously shown that paternal transmission of the deletion leads to an absence of *Igf2* specifically in the endocrine cells of the placenta, indicating that *Igf2* is imprinted in placental endocrine cells.^18^ In litters where all the placentas were UE, IGF2 protein abundance was, as expected, drastically reduced in the placental Jz, as assessed by immuno-fluorescence (Figure 1C). *Igf2* was also reduced at mRNA level in dissected Jz samples (a degree of contamination with Lz explaining the residual levels observed in mutants; Figures S1E and S1F). Using this strategy, we principally aimed to measure the impact of loss of *Igf2* from the placental endocrine cells on maternal metabolism and its consequences for fetal development.

Exclusive loss of *Igf2* in the endocrine cells of the placental Jz led to reduced litter size and symmetrical fetal growth restriction, with a 9% reduction in the average fetus weight at gestational day (GD)16, and an increased percentage of fetuses below the 10th centile (Figures 1D–1G and S2A–S2F). However, placental weight, Jz volume, and Jz cellular composition were unaltered between the two experimental groups, although placental efficiency was significantly reduced in the UE group (Figures 1H–1J and S2G).

Because the observed fetal growth restriction was not attributable to gross defects in placental morphology, and glucose is the principal substrate driving fetal growth and energy metabolism, we then measured maternal-fetal parameters of glucose homeostasis. Fetal glycemia was significantly lower in UE fetuses than controls by 33%, and *in vivo* placental glucose clearance was reduced by 24% (Figures 1K–1M). Once fetal weight was accounted for, no change in fetal glucose accumulation was observed (Figure 1N) and expression of glucose transporters (*Slc2a1* and *Slc2a3*) in the dissected placental Lz was unchanged (Figure S2H). Taken together, these findings suggest that placental endocrine *Igf2* (henceforth referred to as placental Jz-*Igf2*) regulates fetal growth through changes in fetal glucose acquisition.

### Loss of placental Jz-*Igf2* results in impaired maternal insulin resistance response

**Figure 2:**
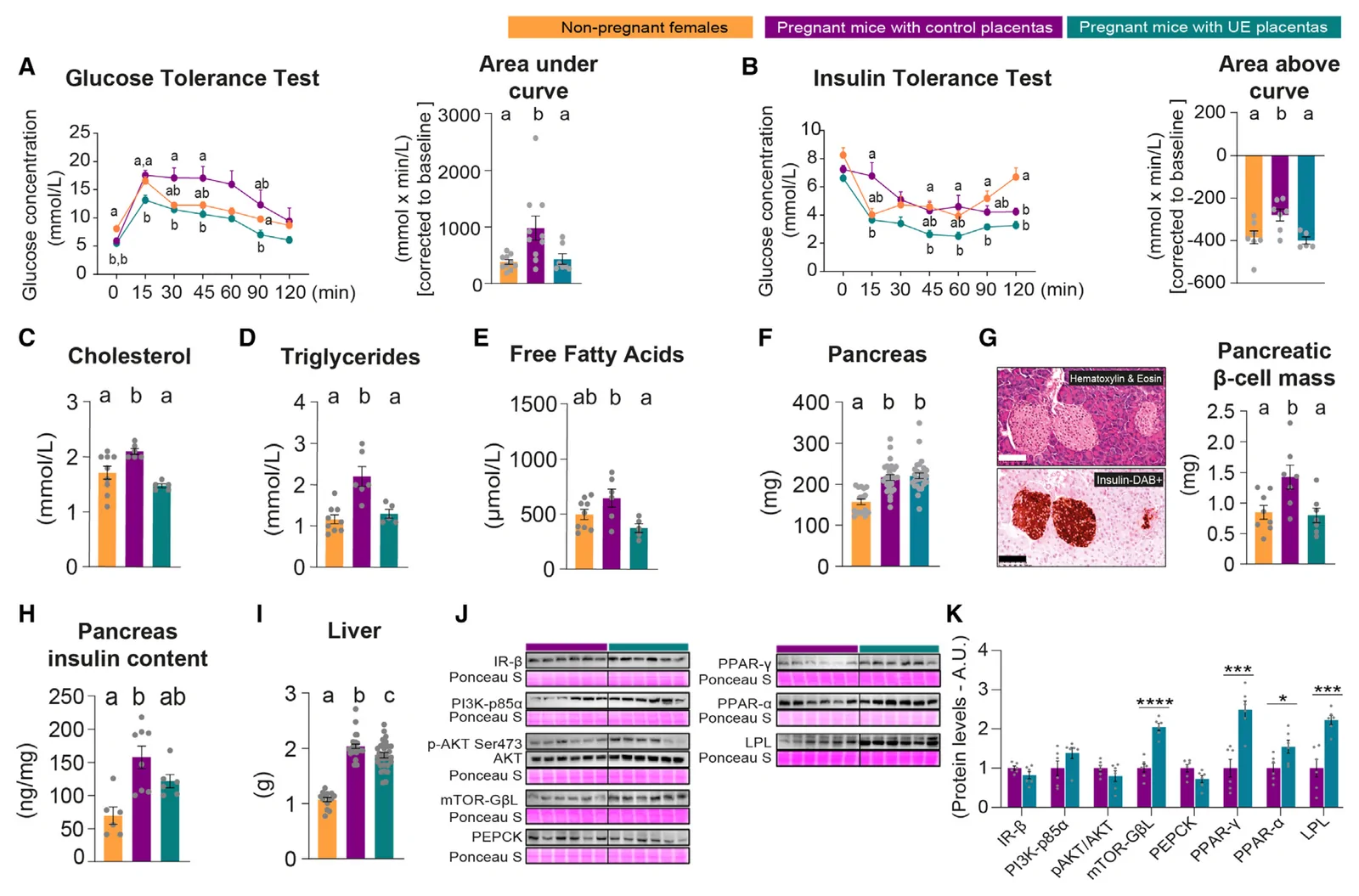
Loss of *Igf2* in the placental endocrine cells impairs maternal metabolic adaptations to pregnancy (A and B) Intraperitoneal glucose and insulin tolerance tests performed on day 16 of gestation. Right panels show area under glucose curve and area above insulin curve (n = 5–10 dams/group/test), respectively. (C–E) Concentrations of cholesterol, triglycerides, and free (non-esterified) fatty acids in maternal circulation (n = 5–9 dams/group/test). (F) Absolute pancreas mass (n = 16–29 dams/group). (G) Representative histological staining of pancreatic β cell islets (insulin-containing cells are 3,3′-diaminobenzidine [DAB] positive) and quantitated pancreatic β cell mass (n = 7–8 dams/group). Scale bar represents 100 µm. (H) Pancreatic insulin content (n = 6–8 dams/group). (I) Absolute liver mass (n = 14–30 dams/group). (J and K) Immunoblots with their respective Ponceau staining as loading control and relative levels of proteins involved in glucose and lipid metabolism in maternal liver (n = 6 dams/group). Data are presented as mean ± SEM and analyzed with Mann-Whitney tests, Student t tests or one-way ANOVA with Tukey post-hoc tests for mean comparisons, as appropriate. Glucose tolerance test (GTT) and insulin tolerance test (ITT) data were analyzed by two-way ANOVA with repeated measures. *p < 0.05;***p < 0.001; ****p < 0.0001. Different letters (a, b, and c) indicate significant differences between groups by one-way ANOVA with Tukey post-hoc tests for mean comparisons. See also Data S1.

A major mechanism by which adequate levels of glucose are delivered to the fetus includes the transient state of maternal insulin resistance that occurs naturally during pregnancy.^19^ *In vivo* metabolic tolerance testing revealed that dams carrying UE litters failed to become glucose intolerant and insulin resistant (Figures 2A and 2B). Glucose tolerance and insulin sensitivity thresholds were instead at similar levels to non-pregnant females. Consistent with their retention of insulin sensitivity, dams with UE litters also did not increase their circulating levels of cholesterol, triglycerides, and free fatty acids as seen in pregnant controls, thus maintaining similar levels to those observed in non-pregnant females (Figures 2C–2E).

Maternal insulin resistance is normally compensated by an increase in pancreatic insulin secretion, which is achieved at least, in part, by an expansion of the b cell mass during pregnancy.^20,21^ Although maternal pancreas weight increased as it should in pregnancy, pancreatic b cell mass expansion failed to occur in UE dams, who displayed a similar pancreatic b cell mass size to non-pregnant females (Figures 2F and 2G). Moreover, pancreatic insulin content also increased to a lesser extent in dams with UE litters (UE values were intermediate between non-pregnant and pregnant dams with control litters; Figure 2H).

The liver, which is vital for whole body metabolism and is an insulin-responsive organ important for whole body glucose and lipid handling, did not fully increase in weight in dams with UE litters during pregnancy (Figure 2I). Lipid handling proteins—LPL, an enzyme that mediates the clearance of lipids by hydrolyzing them so that they can be rapidly taken up by tissues,^22^ and PPARs (g,a), important for fatty acid metabolism,^23^ and glucose homeostasis^24^—were all increased in the liver of UE dams (Figures 2J and 2K). mTORC1 protein GbL, which is activated by insulin,^25,26^ was also significantly elevated in the liver of UE dams (Figures 2J and 2K). Although there was an absence of deregulation of canonical markers of hepatic insulin signaling (i.e., insulin receptor [IR]/AKT/PI3K; Figures 2J and 2K), these findings are consistent with the inability to develop insulin resistance and increase circulating lipids in pregnancy. Together, these data suggest that placental Jz-*Igf2* controls maternal metabolic adaptations, supporting fetal nutrient acquisition and fetal growth.

**Figure 3:**
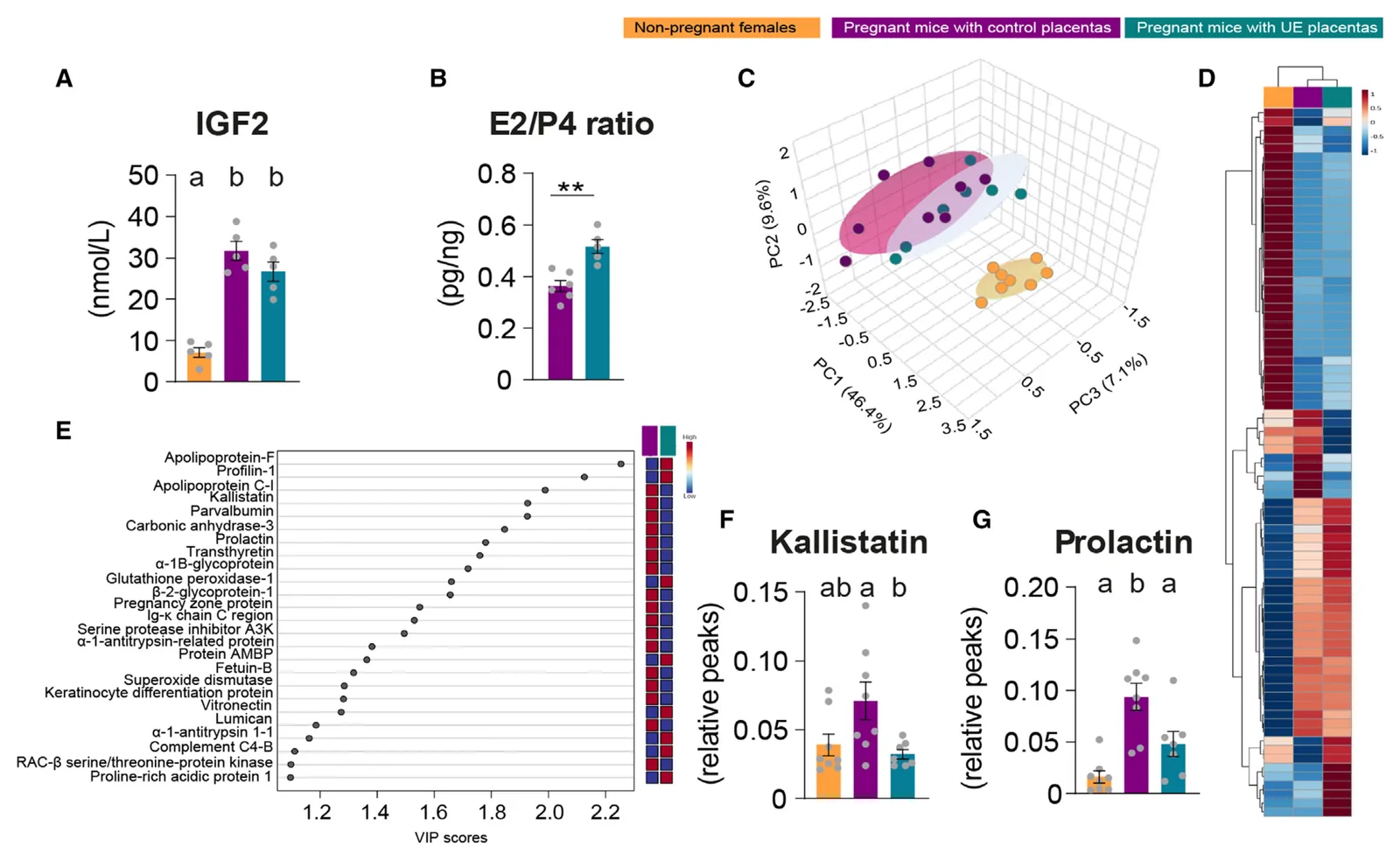
Loss of *Igf2* in placental endocrine cells alters the abundance of circulating proteins and hormones in the maternal circulation (A and B) Circulating concentrations of IGF2 in maternal plasma (n = 5/group) and ratio of estradiol (E2) to progesterone (P4) in maternal plasma detected by ELISA (n = 5–6/group). (C) 3D principal-component analysis (PCA) plot representing the first three components that capture the highest sources of data variation between the three groups of maternal plasma proteins measured by liquid chromatography mass spectrometry (LC-MS/MS) (n = 7–8/group). (D) Hierarchical clustering heatmap of the 80 proteins quantified by LC-MS/MS used for PCA. Data are shown as average values per group, with dendrogram for samples shown on top of the heatmap and the dendrogram of proteins on the left side. (E) Forest plot of control versus UE maternal plasma, depicting the top 25 proteins ranked based on their highest VIP (variable importance in projection) scores. These are the proteins contributing the most to the sub-separation observed in the PCA plot between UE and control samples as informed by partial least-squares discriminant analysis. Colored boxes on the right indicate the relative abundances of proteins in each group. (F and G) Relative levels of kallistatin and prolactin detected in maternal plasma by LC-MS/MS (n = 7–8/group). Data are presented as mean ± SEM with individual data points shown. Student t tests for two groups analysis or one-way ANOVA with Tukey post-hoc tests for mean comparisons were performed, as appropriate.**p < 0.01. Different letters (a and b) indicate significant differences between groups by one-way ANOVA. See also Data S1 and Table S1.

To elucidate the mechanisms that led to failure in acquiring the pregnancy-associated insulin resistance and b cell mass expansion in UE dams, we first investigated the levels of selected maternal circulating hormones and performed unbiased quantitative liquid chromatography-tandem mass spectrometry (LC-MS/MS) on GD16 maternal plasma. We found no difference in IGF2 concentrations, which are extremely low in adult mice, between control and UE pregnant dams (Figure 3A). Although IGF2 is increased ~3-fold in pregnancy compared with non-pregnant state, these findings ruled out a direct contribution of circulating IGF2 to the metabolic phenotype observed in UE dams. Circulating estradiol to progesterone ratio was greater in dams carrying UE litters compared with dams carrying control litters (Figures 3B, S3A, and S3B), suggesting perturbations in steroid hormone production. Based on the overall profile of 80 proteins reliably quantified out of the total of 180 detected by proteomics analysis in the maternal plasma, we observed a clear segregation between the samples according to the pregnancy status, with more subtle differences in clustering between pregnant dams carrying UE compared with control litters (Figures 3C–3E; Table S1). Forest plot of the top 25 proteins highlighted the key proteins that accounted for the segregation of proteins dysregulated between pregnant UE and control dams (Figure 3E). Of note, two proteins in the circulation were significantly decreased in UE relative to control dams, namely, prolactin (encoded by the *Prl* gene), a hormone with roles in metabolic regulation, including insulin sensitivity and b cell proliferation,^20^ and kallistatin (encoded by *Serpina4*), a serine proteinase inhibitor that is elevated in the circulation of people with diabetes and correlates with lipid levels^27^ (Figures 3F and 3G). The relative abundances of six additional proteins in the circulation of UE dams were intermediate between non-pregnant and pregnant control dams, including CFB (Figures S3C–S3H), which is involved in glucose-insulin handling.^28^ Other proteins such as VTNC, which is correlated with hepatic insulin sensitivity,^29^ were significantly elevated in the circulation of UE dams compared with non-pregnant (pregnant controls were intermediate between non-pregnant and UE dams; Figures S3I–S3K). Taken together, these findings show that the levels of several proteins/hormones in the maternal circulation are directly or indirectly controlled by placental Jz-*Igf2* and may drive changes in maternal metabolism.

### Placental factors contributing to maternal metabolic dysfunction in UE mice

**Figure 4:**
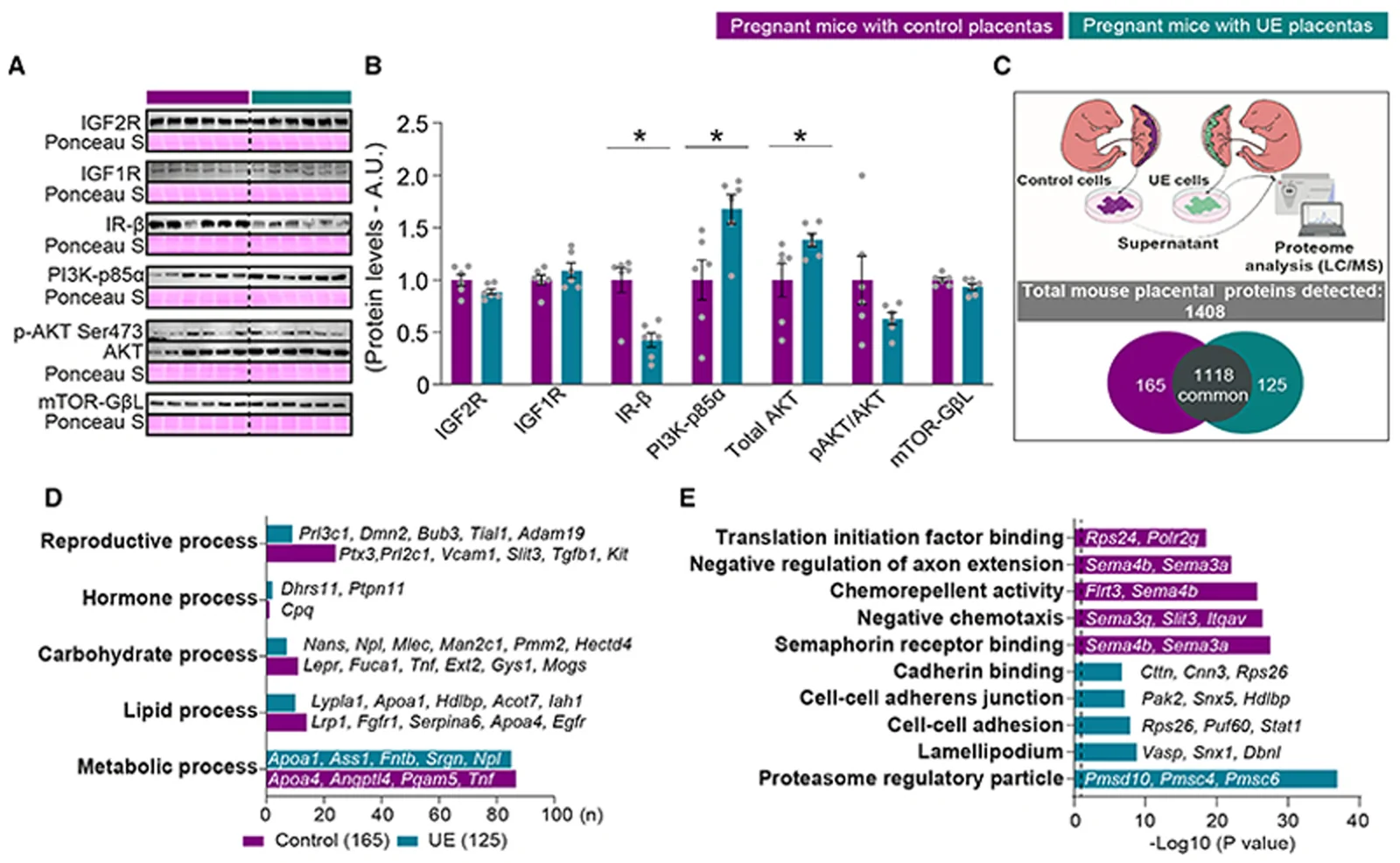
Placentas with loss of *Igf2* in the endocrine cells show abnormal protein signaling and altered function (A and B) Immunoblots with their respective Ponceau staining as loading control and relative levels of proteins involved in metabolism and cell growth in placental Jz lysates. Data were analyzed with Mann-Whitney or Student t tests and presented as mean ± SEM, *p < 0.05. (C) Schematic of experimental design for placental proteomics with the number of proteins detectable by LC-MS/MS from control and UE placental cell culture experiments (n = 3 litters/group). Liquid chromatography mass spectrometry (LC-MS) was performed on supernatants derived from conditioned medium of cultured primary cells from dissected Jz of control and UE placentas. Note: separated primary cultures are not pure; hence, there is potential contamination of Lz cells, which may limit the detection of Jz proteins. (D) Relevant pathways identified by UniProt analysis of proteins uniquely present in the control and UE secretomes. On the x axis, n, number of proteins for each gene ontology term. To facilitate the visualization of the graph, gene names were used instead of protein names. (E) Top 5 scoring biological processes obtained from DAVID annotations (further information can be found in Table S2). See also Data S1 and Table S2.

Consistent with the lack of cellular composition changes and morphological defects within the UE placenta, protein levels of IGF receptors (IGF2R and IGF1R) and downstream signaling proteins that are the transducers of IGF2 for cell proliferation (phosphorylated levels of AKT at serine 473 and mTOR-GbL) were unaltered compared with controls (Figures 4A and 4B). Interestingly, abundances of insulin receptor (IR-b), PI3K regulatory subunit p85a and total AKT were disrupted, suggestive instead of impaired metabolic responses in UE placentas (Figures 4A and 4B).

To explore the impaired UE placental metabolic response further and to identify placental secreted proteins mediating maternal metabolic dysfunction in UE dams, we prepared bulk primary cultures of placental endocrine cells from control and UE litters and analyzed the conditioned media using qualitative LC-MS/MS (Figure 4C). A total of 1,408 proteins in the conditioned media were detected (Figure 4C), including known secreted proteins—placental lactogen/prolactin family members (e.g., PRL3B1, PRL8A8, and PRL7A1) and pregnancy-specific glycoproteins (e.g. PSG16, PSG21, and PSG23) and IGFBPs (e.g., IGFBP6 and IGFBP7). Additionally, overlapping analysis of the 1,408 proteins detected in the placental secretome (regardless of placenta genotype) with the 180 proteins detected in the maternal plasma, resulted in the identification of 80 placental proteins contributing to the levels measured in the maternal circulation (e.g., SERPINA6, CP, ITIH4, and LIFR; Table S2). Moreover, several of the proteins detected in the placental secretome displayed a different pattern of expression in the pregnant UE, relative to the non-pregnant and pregnant control dams (e.g., semaphorins, prolactins, SERPINAs, VTNC, UBE2N, GPX1, and APOs; Figures 4D and S3), suggesting that the placenta, directly or indirectly, contributed to their differential levels in the maternal circulation (Table S2).

UniProt analysis, which focused on the 290 proteins that were detected in one group but not the other (i.e., 125 unique proteins in UE and 165 unique proteins in controls), identified biological terms related to metabolic processes that comprised 52% and 68% of the unique proteins in the control and UE secretomes, respectively (Figure 4D; Table S2). UniProt analysis also revealed additional terms, including hormone/estrogen biosynthetic and reproductive processes (Table S2). Of note, several proteins have been implicated in modulating insulin resistance/sensitivity (e.g., TNFA^30^ and semaphorins,^31^ absent in UE but present in controls), with other proteins showing a qualitative gain or loss in UE, which include key regulators of lipid homeostasis (e.g., FGFR1^32^ and APOA4^33^), glucose metabolism (e.g., LEPR^34^ and FUCA1^35^), and b cell proliferation (e.g., PRL2C1^20^ and FGFR1^36^). Finally, DAVID functional analysis, which was performed with the aforementioned 290 proteins, identified a total of 41 gene ontology (GO) terms altered in response to UE, including processes related to translation, cell adhesion, cell migration, extracellular exosome, and semaphorin receptor binding (Figure 4E; Table S2).

**Figure 5:**
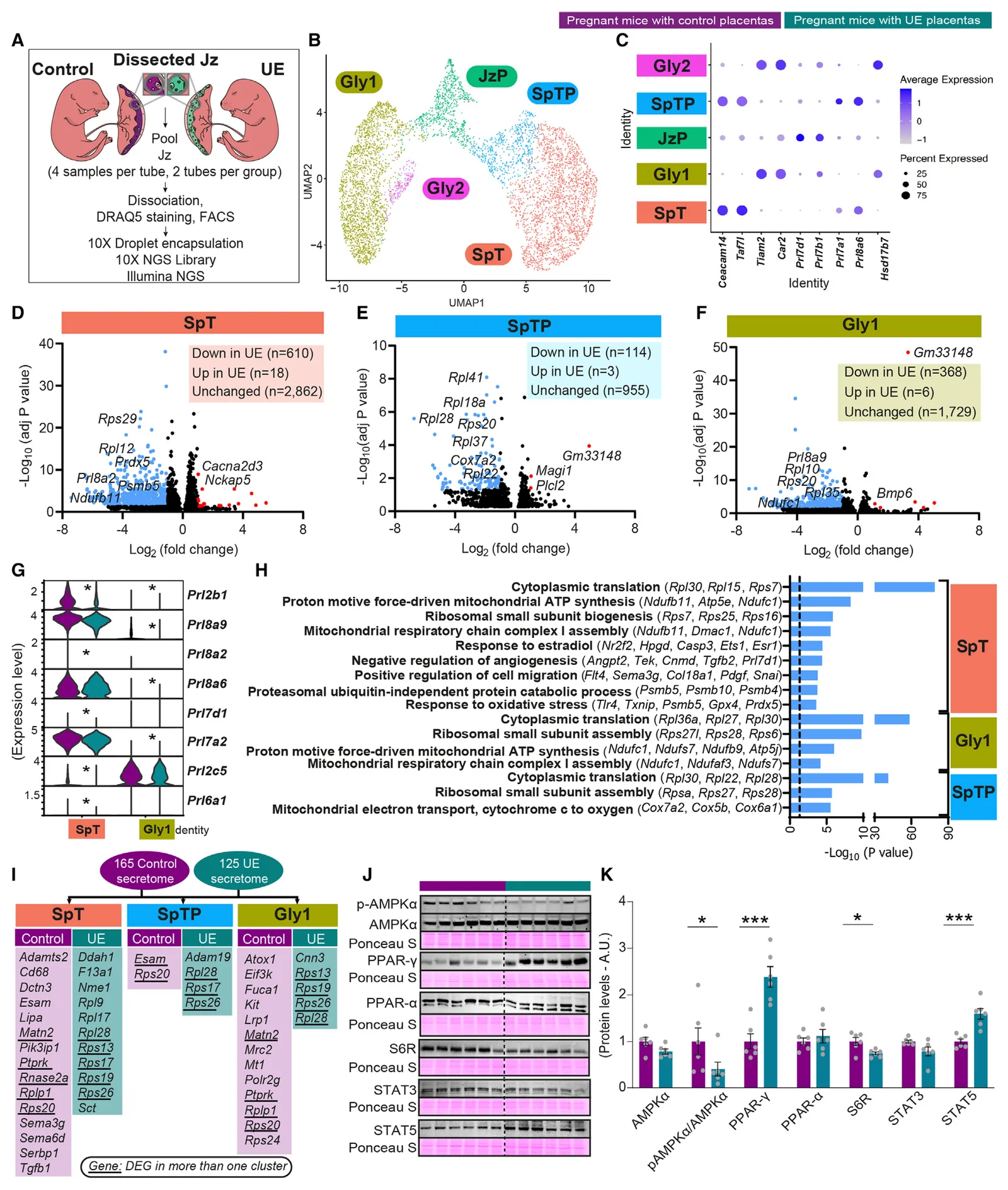
Loss of *Igf2* in placental endocrine cells alters placental-cell-specific transcriptomics and induces changes in mitochondrial and ribosome homeostasis (A) Schematic illustration of the approach taken for snRNA-seq. Two biological replicates per group were analyzed. Each replicate contained micro-dissected Jz samples from 2 females and 2 males (5–7 litters/group). (B) Uniform manifold approximation and projection (UMAP) plot showing Jz cellular populations. Each dot represents a single cell and is colored by their cell cluster assignment (Gly1, Gly2, JzP, SpTP, and SpT). Further information shown in Figure S4. (C) Dot plot showing the expression of genes characteristic of each placental endocrine cell type. (D–F) Volcano plot showing differentially expressed genes (DEGs) between UE and control cell populations (further information in Figure S5 and Table S3). (G) Violin plots showing the expression levels of differentially regulated prolactin genes in SpT1 and Gly1 clusters. *indicates statistical differences (p < 0.05). (H) Top scoring biological processes enriched in DEGs for Jz clusters SpT, Gly1, and SpTP. (I) Overlap of the datasets generated from the placental secretome (proteins that were detected in one group but not the other: 125 unique proteins in UE and 165 unique proteins in controls) and the DEGs detected by snRNA-seq. Genes underlined belong to more than one cluster of Jz cells. (J and K) Immunoblots with their respective Ponceau staining as loading control and relative levels of proteins involved in metabolism and other cell functions in placental Jz (n = 6/group). Data were analyzed with Mann-Whitney or Student t tests and presented as mean ± SEM. *p < 0.05, ***p < 0.001. See also Data S1 and Table S3.

To further identify autocrine/paracrine mechanistic actions of IGF2 that ultimately change the maternal metabolic profile, single nuclear RNA sequencing (snRNA-seq) of placental endocrine cells from control and UE litters was undertaken (Figure 5A; Table S3). According to previous work,^37^ five main endocrine cell populations were identified, and these showed reduced *Igf2* mRNA levels overall in UE (Figures 5B, 5C, and S4). Each endocrine cell population expressed a unique set of transcription factors (Figure S5A). Differential expression analysis demonstrated a cell-type-specific impact in response to UE, with three of the cell clusters (SpT [spongiotrophoblast cells], SpTP [spongiotrophoblast precursor cells], and Gly1 [glycogen cells type 1]) showing the highest number of differentially expressed genes (DEGs) (Figures 5D–5F, S5B, and S5C). DEGs were predominately downregulated and included several *Prls* that showed cell-specific changes (Figure 5G). DAVID analyses of DEGs highlighted translation, ribosome biosynthesis, response to estradiol, cell migration, and mitochondrial respiratory capacity as the most over-represented GO terms in SpT, SpTP, and Gly1 cell clusters (Figure 5H). Several of the DEGs identified in these endocrine cell types were among those detected at the protein level in the lists unique to control or UE placental secretomes (e.g., LRP, semaphorins, and RPS family members; Figures 5I and S5D). Consistent with DAVID analyses, activation of the energy deficit sensor,^38,39^ AMPK, and total ribosomal protein S6R were reduced in the placental Jz with UE (Figures 5J and 5K). PPARg and STAT5 were increased in the UE Jz, which may reflect a compensatory response to mitochondrial and prolactin deficiency.^40,41^ Collectively, these findings show that placental Jz-*Igf2* impacts the synthesis of proteins/hormones and highlight a potential set of proteins secreted from the placenta into the maternal circulation that could be the key mediators of the maternal metabolic maladaptations detected in the UE dams.

### *In utero* placental programming of metabolic dysfunction observed in adult offspring

**Figure 6:**
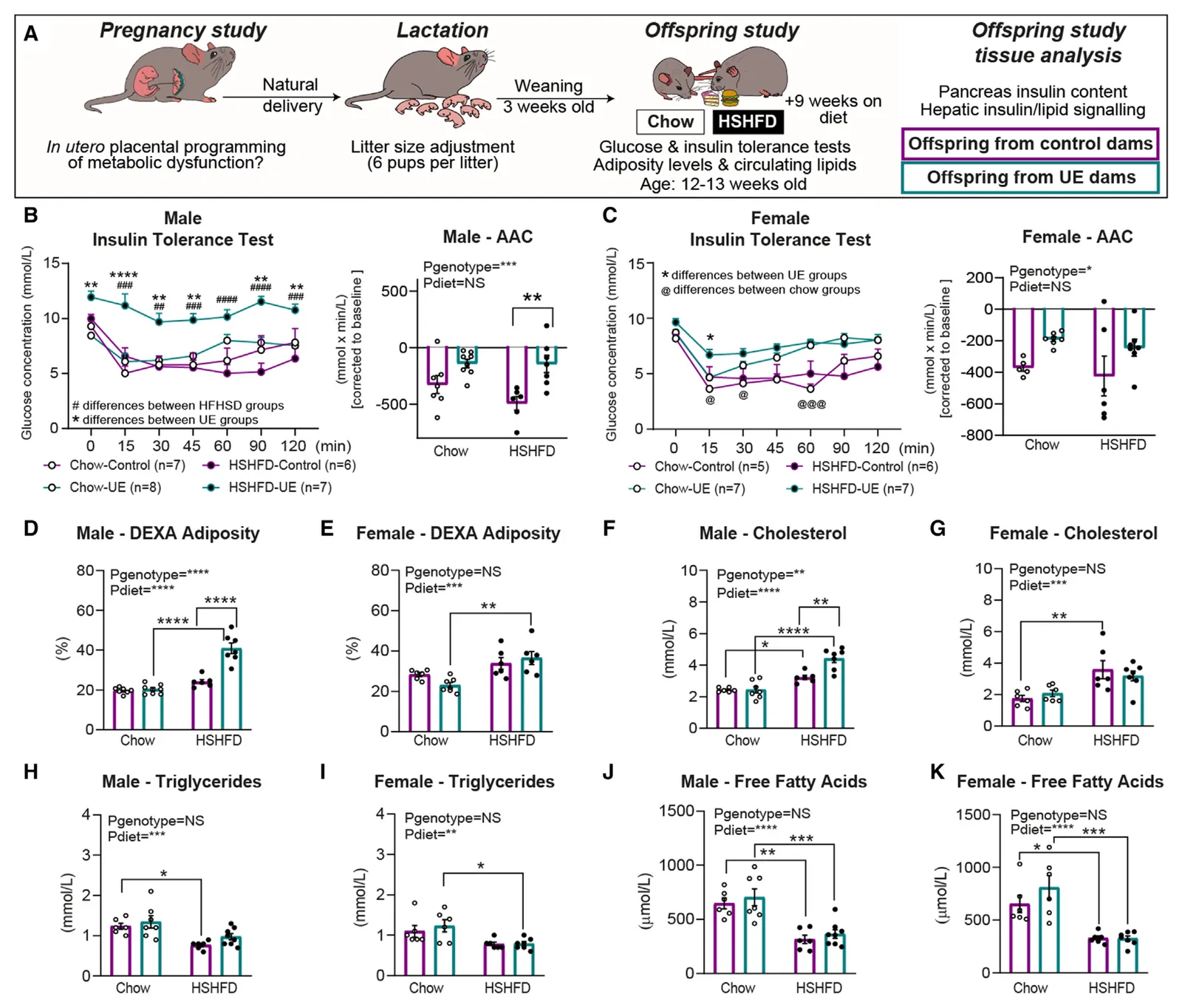
Offspring with placental endocrine cell *Igf2* loss have impaired insulin tolerance independent of diet and increased adiposity and altered circulating lipids on a high sugar, high fat diet (HSHFD) (A) Schematic of experimental design for offspring follow-up. (B and C) Intraperitoneal insulin tolerance tests were performed on male and female offspring on chow and HFHSD at 12 weeks of age. Panels on the right show area above insulin curve (AAC) (n = 5–8 mice/group/sex; each dot represents a different litter). (D and E) Adiposity as measured by dual-energy X-ray absorptiometry (DEXA) scans in male and female offspring on chow and HSHFD at 13 weeks of age (n = 6–8 mice/group/sex; each dot represents a different litter). (F–K) Circulating concentrations of cholesterol, triglycerides, and free fatty acids in male and female offspring on chow and HFHSD at 13 weeks of age (n = 6–8 mice/group/sex; each dot represents a different litter). Data are presented as mean ± SEM with individual data points shown. Two-way ANOVA was performed with Tukey post-hoc test for mean comparisons. *p < 0.05; **p < 0.01;***p < 0.001; ****p < 0.0001. Different symbols (*, @, and #) indicate significant differences between groups by two-way ANOVA. See also Data S1.

Direct evidence that perturbation of placental imprinting can program offspring for metabolic disease in later life is lacking. Taking advantage of our unique placental-specific genetic manipulation, we evaluated the metabolic health and body composition of UE and control litters that were fed from weaning, either a normal chow diet or a high sugar-high fat diet (HSHFD) (Figure 6A). There was no difference in the weight of the offspring during lactation and following weaning between control and UE litters, with food intake after weaning comparable to their diet-matched control counterparts (Figure S6).

Notably, UE offspring were insulin resistant, regardless of the postnatal diet or sex, when compared with controls (Figures 6B and 6C). However, glucose tolerance was unaffected in UE offspring, fed with chow or HSHFD (Figures S7A and S7B). Although there was no difference in adiposity between UE and control offspring on a chow diet, both UE male and female offspring showed a greater gain of adiposity on a HSHFD (~20% for UE for both sexes and ~5% and 10% for male and female controls; Figures 6D and 6E). Furthermore, UE male offspring were approximately two times fatter (% adiposity) than control males when fed a HSHFD. Circulating lipid (cholesterol, triglycerides, and free fatty acids) levels were not different between UE and control offspring on a chow diet, regardless of sex and genotype (Figures 6F–6K). HSHFD significantly increased circulating cholesterol in the offspring. However, this was exacerbated in UE males who had elevated concentrations compared with HSHFD control males (Figure 6F). The HSHFD also reduced circulating triglycerides, but this was less pronounced in UE male offspring who had increased concentrations compared with HSHFD-fed control males (Figure 6H). In addition, the reduction in circulating triglycerides was significant for HSHFD-fed UE, but not in control female offspring (Figure 6I).

**Figure 7:**
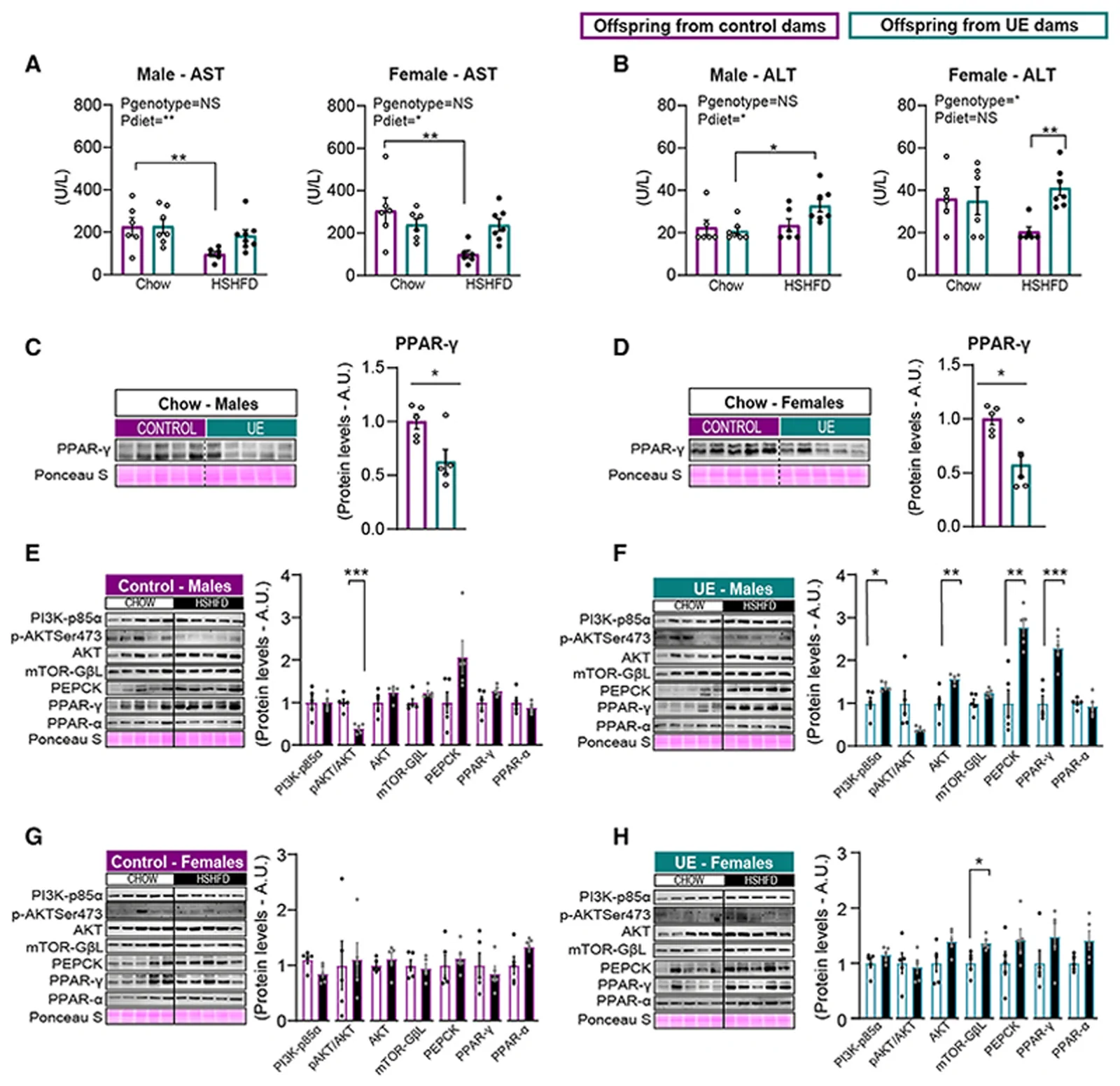
Offspring with placental Jz *Igf2* loss have abnormal hepatic function and altered levels of proteins involved in metabolism (A and B) Circulating levels of aspartate alanine transferase (AST) and alanine aminotransferase (ALT) in male and female offspring on a chow or HSHFD at 13 weeks of age (n = 6–8 mice/group/sex; each dot represents a different litter). (C and D) Immunoblots of PPARg with their respective Ponceau staining as loading control and relative levels in liver from male and female offspring on a chow diet at 13 weeks of age (n = 5 mice/group/sex; each dot represents a different litter). (E–H) Immunoblots and a representative Ponceau staining as loading control and relative levels of proteins involved in glucose and lipid metabolism in liver from male and female offspring on a HSHFD at 13 weeks of age (n = 5 mice/group/sex; each dot represents a different litter). Data are presented as mean ± SEM with individual data points shown. Two-way ANOVA was performed with Tukey post-hoc test for mean comparisons (liver weights and AST and ALT concentrations). Western blots were analyzed by Mann-Whitney or Student t-tests. *p < 0.05, **p < 0.01, ***p < 0.001. See also Data S1.

To elucidate the factors that underpin the observed metabolic phenotype, we then examined two offspring metabolic organs, the pancreas and the liver. Although HSHFD decreased pancreatic weight overall, there was no effect of genotype (Figure S7C). No differences were observed in the pancreatic insulin content independently of the genotype or postnatal diet (Figure S7D). Regarding the liver, although the intrauterine exposure to UE placentas (P_genotype_) had no effect on liver weight, HSHFD increased liver weight of female but not of male offspring (Figure S7E). Circulating levels of aspartate aminotransferase (AST), a marker of hepatic function, were particularly lowered as a result of a HSHFD in control offspring regardless of sex, an effect that was not seen in UE offspring fed the HSHFD (Figure 7A). In contrast, the levels of alanine aminotransferase (ALT), another hepatic function marker, were increased by a HFHSD in UE males but not in control offspring (Figure 7B). Furthermore, UE females showed increased ALT compared with controls when they were both fed the HFHSD (Figure 7B). Therefore, these data show that nutritionally induced changes in postnatal hepatic function are programmed by placental Jz-*Igf2* in a sex-dependent manner.

Molecular analyses of offspring hepatic insulin signaling and lipid handling proteins (Figures 7C–7H and S7F–S7K) revealed that on a chow diet, PPARg protein, which when low is associated with insulin resistance and hyperlipidemia in aged mice,^42^ was reduced in UE offspring, regardless of sex, compared with their respective controls (Figures 7C and 7D). Levels of other lipid handling or insulin signaling proteins on a chow diet were unaltered (Figures S7F and S7G). However, we observed altered levels of PI3K-p85a, total AKT, PEPCK, and PPARg in UE males on HSHFD compared with chow but not in male control groups (Figures 7E and 7F). LPL levels were increased in both HSHFD-fed UE male and control groups (Figures S7H and S7I), with a significant downregulation in AKT activation only observed with a HSHFD in control offspring (Figures 7E and 7F). These molecular changes are consistent with our findings that UE male offspring may be more, and differentially susceptible to, metabolic disturbances on an obesogenic diet (Figures 6B–6I, 7A, and 7B). In UE female offspring, we observed fewer and different changes with a HSHFD compared with chow and their control groups (Figures 7G, 7H, S7J, and S7K). We found that mTOR-GbL levels, which were not affected in female controls by a HSHFD, were increased in the HSHFD-fed UE females compared with chow (Figure 7G).

Overall, these data demonstrate that placental Jz-*Igf2* induces programming effects in the adult offspring. Namely, both UE female and male offspring are programmed for insulin resistance that is partly explained through hepatic molecular changes and increased predisposition to a greater gain of adiposity on an obesogenic diet.

## Discussion

From an evolutionary point of view, insulin resistance during pregnancy is designed to limit maternal glucose utilization by target tissues and thereby shunt an adequate amount of supply to the developing fetus. In humans, there is a linear relationship between maternal glucose tolerance during pregnancy and birthweight.^43^ The mechanisms that render the mother’s tissues less insulin sensitive are poorly understood, but are believed to be caused, at least in part, by placental hormones.^2,44^ To our knowledge, the role of the placenta, or of a single hormone, in this process has not been directly demonstrated. Here, we have shown in the mouse that IGF2, a peptide with structural similarities to insulin and produced in placental endocrine cells, is required to establish the insulin resistance state in pregnancy.

Our data indicate that loss of *Igf2* from the endocrine layer of the mouse placenta alters the secretion of key signaling proteins into the maternal circulation, which, in turn, leads to an impaired maternal endocrine and lipid profile. This is related to defects in placental endocrine cell mitochondrial energetics and ribosome/translation capacity, as suggested by the down-regulation of key genes identified by our snRNA-seq approach. Given that the gross placental stereological assessment showed no overt differences in endocrine cell density in UE placentas, changes in signaling probably occurred through autocrine and paracrine routes via the IR and downstream pathway proteins (PI3K) for IGF2, which are present in individual endocrine cells and were altered in our study. Indeed, prior work highlights an important role for PI3K signaling in the expression of hormones by the mouse placenta.^45–47^ There were also endocrine-cell-specific changes in key placental hormones, including the *Prls* due to *Igf2* loss. Ultimately, these placental events caused by *Igf2* loss render maternal organs more insulin sensitive, with important consequences for fetal development. Our data strongly suggest that the loss of fetal viability, fetal hypoglycemia, and fetal growth restriction observed in our mouse model are caused by insufficient glucose and lipid delivery to the fetus, mainly driven by the higher glucose utilization/uptake by the maternal organs as a result of increased glucose tolerance and insulin sensitivity. The UE placentas are therefore less efficient in maintaining fetal growth trajectories mainly due to the impaired endocrine signaling to the mother (rather than due to functional or morphological defects of the transport epithelium).

Another major finding of our work is that the pancreatic β cell mass in mothers that carry mutant *Igf2* UE placentas fails to expand in response to pregnancy. Pancreatic β cell mass expansion is thought to occur in response to increased insulin resistance in maternal tissues.^48^ Our findings may, therefore, reflect the failure in acquiring the insulin resistance state. We suggest that mutant *Igf2* UE placentas fail to secrete hormones that are necessary to stimulate β cell expansion, such as prolactins, which were also decreased in the circulation of UE dams. Indeed, prior work using litters comprising both control and UE fetuses has reported that *Igf2* deficiency in placental endocrine cells reduces the expression of prolactin genes and disrupts the expression of *Cyp17a1*, which would favor the production of estrogen over progesterone by the placenta.^18^ Herein, we found altered secretion or abnormal circulating levels of previously described regulators of insulin sensitivity during gestation, namely, estradiol/progesterone ratio, prolactin, and TNFA. As novel mediators, we found reductions in circulating levels of serine protease inhibitors, including kallistatin. In the liver, the observed increases in PPARs, mTOR, and LPL abundance may contribute to the reduction in circulating lipids.

Imprinted *Igf2* expression in placenta endocrine cells may have evolved as a strategy to mobilize nutrients to the growing fetus, in line with the concept of imprinted genes having evolved due to conflict.^4^ Indeed, pregnancy complications such as pre-eclampsia and gestational diabetes can be seen as conflict-based manipulation systems going wrong, whereby increased blood pressure and glucose levels, respectively, mainly serve the interests of the fetus rather than that of the mother.^49^ Based on our targeted loss-of-function results, we suggest that Jz-*Igf2* is a major modulator of maternal metabolic adaptations that ensures mobilization of nutrients, namely, glucose and lipids, to the fetus by inducing insulin resistance and enhancing maternal glucose and lipid availability, which have implications for adult health. This proposal is further supported by circumstantial evidence collected in mice and human studies,^50^ including from human imprinting disorders such as Beckwith-Wiedemann Syndrome (BWS), an overgrowth syndrome, and Silver-Russell Syndrome (SRS), a growth restriction syndrome. In a mouse model of BWS, dams that carry overgrown offspring with increased *Igf2* and reduced levels of the maternally expressed *H19* gene in most fetal cells develop maternal hyperglycemia and glucose intolerance.^51^ Our past work in litters of mixed genotype with either Jz deletion of ICR1 that results in increased *Igf2* and reduced *H19* expression or Jz deletion of *Igf2* in the placenta suggest that changes in placental endocrine capacity and altered signaling may contribute to the observed phenotypes.^18,52^ Metabolic phenotypes in offspring, such as neonatal hyperinsulinemia and hypoglycemia, have also been reported in subgroups of patients with BWS and SRS who show increased *IGF2* or low *H19* and low *IGF2* or increased *H19* expression levels in tissues,^53,54^ respectively. BWS-associated hyperinsulinism occurs due to β cell dysfunction that can result in transient and prolonged hypoglycemia.^55^ Furthermore, patients with BWS have an increased risk of cancers/tumors in early childhood (e.g., Wilms tumor, hepatoblastoma, adrenocortical carcinoma, or pancreatoblastoma).^56^ However, relatively less information about patients with BWS and SRS relates to the later adult rather than the perinatal/early neonatal periods. The problems reported in adult patients with BWS suggest a cumulative higher risk of cancer or arose subsequent to pediatric issues,^57^ whereas SRS is linked with poorer metabolic health, including hypercholesterolemia, glucose intolerance, and insulin resistance in adulthood.^58,59^ Nonetheless, because we only assessed the effects in our model up to 12–13 weeks of age, which corresponds to young adulthood, whether the observed metabolic deficits in UE offspring persist in later life, and if there are potential changes to the germ line that may affect future generations, remain to be investigated in future studies.

Currently, there are no data to support a role for placental imprinting in fetal programming of metabolic disease, mainly due to the lack of appropriate, specific genetic tools. We found that male and female offspring with *Igf2* deficiency in placental endocrine cells become insulin resistant and have elevated adiposity, with this effect stronger particularly under conditions that favor the mismatch of programmed adaptations *in utero* (i.e., intrauterine growth restriction due to compromised nutrient supply, with a postnatal obesogenic environment). This programming of insulin resistance was accompanied by specific changes in the abundance of key insulin-signaling proteins in the liver that were most pronounced for males. However, further work is required to study how offspring phenotype is programmed in our UE model.

In conclusion, this study provides experimental evidence for an intrinsic fetal manipulation system, which operates in the placenta to modify maternal metabolism and nutrient partitioning to the fetus, with implications for the metabolic health and disease risk of offspring in later life.

## Limitations of the study

Our study has a few limitations. Longitudinal studies, to be performed at earlier and later stages of pregnancy, will be required to understand the full extent by which the Jz-*Igf2* mutant placenta modifies maternal metabolism via impaired secretion of proteins (i.e., studies focusing on placental-derived secreted proteins—e.g., prolactin-like proteins and semaphorins, as a number of those were identified as differentially expressed at the transcriptome level in this study). The molecular basis of why mothers become insulin sensitive and offspring are programmed for metabolic dysfunction will require whole-body physiology studies to identify the key metabolic organ targets followed by comprehensive molecular analyses on those tissues.

## Star★Methods

### Key Resources Table

**Table:** 

REAGENT or RESOURCE	SOURCE	IDENTIFIER
Antibodies		
Insulin	Cell Signaling	RRID:AB_659820
Insulin receptor beta	Cell Signaling	RRID:AB_2249166
PI3K-p85 alpha	Millipore	RRID:AB_310069
Phospho-Akt (Ser473)	Cell Signaling	RRID:AB_2315049
AKT	Cell Signaling	RRID:AB_329827
GβL (86B8)	Cell Signaling	RRID:AB_823685
PEPCK	Santa Cruz	RRID:AB_10610383
Lipoprotein lipase	Abcam	RRID:AB_446221
PPAR alpha	Abcam	RRID:AB_303844
PPAR gamma	Santa Cruz	RRID:AB_628115
Phospho-AMPK (Thr172)	Cell Signaling	RRID:AB_331250
AMPK	Cell Signaling	RRID:AB_10624867
STAT3	Millipore	RRID:AB_10807169
STAT5	Cell Signaling	RRID:AB_2798908
S6 Ribosomal protein	Cell Signaling	RRID:AB_2146239
IGF-II Receptor	Cell Signaling	RRID:AB_2753208
IGF-I Receptor	Cell Signaling	RRID:AB_10950969
IGF-II	R&D Systems	RRID:AB_354449
Alexa Fluor 488	Jackson ImmunoResearch	RRID:AB_2340430
Sheep Anti-Mouse IgG	GE Healthcare	RRID:AB_772210
Donkey Anti-Rabbit IgG	GE Healthcare	RRID:AB_772206
Goat Anti-Rabbit IgG	Abcam	RRID:AB_954902
Chemicals, peptides, and recombinant proteins		
RIPA buffer	Sigma	R0278
Streptavidin-conjugated peroxidase	Rockland Immunochemicals	S000-03
3,3′-Diaminobenzidine	Abcam	ab64238
Nuclear fast red	Vector Laboratories	H-3403
DAPI	Sigma	D9542
RNeasy Plus Mini Kit	Qiagen	74134
RNeasy Fibrous Tissue Mini Kit	Qiagen	74704
RNeasy Lipid Tissue Mini Kit	Qiagen	74804
RevertAid RT Reverse Transcription Kit	Thermo Fisher Scientific	K1622
SYBR Green JumpStart Taq Ready Mix	Sigma	S4438
Biosol	National diagnostics	LS-310
NTCT-135 medium	Thermo Fisher Scientific	41350026
Pierce BCA Protein Assay Kit	Thermo Fisher Scientific	23225
Nitrocellulose membranes	Biorad	1620115
SuperSigna West Femto Substrate	Thermo Fisher Scientific	34094
Ponceau S Staining solution	Thermo Fisher Scientific	A40000279
OptiPre Density Gradient Medium	Sigma	D1556
Chromium NEXT GEM Single Cell 3’ GEM, Library & Gel Bead Kit	10X Genomics	1000128
Chromium Next GEM Chip G Single Cell Kit	10X Genomics	1000127
^3^H-methyl-D glucose	Perkin-Elmer	NEN NEC-377
Critical commercial assays		
Insulin	Mercodia	RRID:AB_2783837
Estradiol	Cayman Chemical	582251
Progesterone	Cayman Chemical	582601
IGF-II	R&D Systems	RRID:AB_2884002
Free Fatty Acids	Roche	11383175001
Triglycerides	Siemens Healthcare	DF69A
Cholesterol	Siemens Healthcare	DF27
Alanine aminotransferase	Siemens Healthcare	DF143
Alanine transaminase	Siemens Healthcare	DF41A
Deposited data		
Proteomics data, see Table S1 and S2	This paper	N/A
Processed gene expression data rom single nuclear RNA-sequencing (snSeq), see Table S3	This paper	GEO accession number: GSE229514
Experimental models: Organisms/strains		
Mouse Igf2fl/fl	Hammerle et al.^60^	N/A
Mouse Tpbpa^Cre^	Simmons et al.^16^	N/A
Mouse Rosa26mT/mG mice	Muzumdar et al.^61^	RRID:IMSR_JAX:007676
Oligonucleotides		
Primers used for genotyping or quantitative real-time PCR, see Table S4	This paper	Sigma Aldrich (Merck)
Software and algorithms		
ZEN 2009	Carl Zeiss	https://www.zeiss.com/microscopy
ImageJ	NIH	https://imagej.nih.gov/ij/
CAST	Visiopharm	https://visiopharm.com
AxioVision 4.7.2	Carl Zeiss	https://www.zeiss.com/microscopy
UniProt Knowledgebase	Uniprot consortium	https://www.uniprot.org/
DAVID v6.8	LHRI	https://david.ncifcrf.gov/
REViGO	Supek et al.^62^	http://revigo.irb.hr
CellBender/CellRanger	Fleming et al.^63^	N/A
Seurat version 4.1.0	Hao et al.^64^	N/A
ScDblFinder V1.9.12	Germain et al.^65^	N/A
GraphPad Prism 8 software	GraphPad	https://www.graphpad.com/
SAS/STAT software	SAS	https://www.sas.com/en_us/software/stat.html
Perseus	Max-Planck-Institute of Biochemistry	https://maxquant.net/perseus/
Other		
SDS Rat and Mouse No.3 Breeding diet	SDS	N/A
Rodent Diet With 45 kcal% Fat	Research Diets	D12451
Mineral Mix	MP Biomedicals	AIN93G

## Resource Availability

### Lead contact

Further information and requests for resources and reagents should be directed to and will be fulfilled by the lead contact, Prof. Amanda N. Sferruzzi-Perri.

### Materials availability

This study did not generate new unique reagents.

## Method Details

### Mice and genotyping

Mice were housed at the University of Cambridge Animal Facility under a 12/12 dark/light system. All experiments were performed under the U.K. Animals (Scientific Procedures) Act 1986, after ethical approval by the University of Cambridge. Both *Igf2*^*Flox*^ and *Tpbpa*^*Cre*^ mouse lines have been on the C57BL/6J genetic background for more than 10 generations.^16,60^ The genotype of mouse lines was confirmed by PCR analysis of genomic DNA extracted from ear biopsies. Details of the primer sequences for genotyping and about the generation of both genetic lines have been reported previously.^18^ To visualise the Cre recombinase activity and quantify mRNA levels of *Tpbpa* and *Igf2* in adult tissues, *Tpbpa*^*Cre*^ mice were mated with Rosa26mT/mG mice (RRID:IMSR_ JAX:007676),^61^ which were generously provided by Dr Maria Alcolea at the University of Cambridge. For studies of pregnancy physiology and fetal outcomes, virgin female homozygous *Tpbpa*^*Cre*^ mice were mated with male homozygous *Igf2*^*Flox*^ to generate pregnancies where the entire litter had selective loss of *Igf2* in the endocrine cells of the placental junctional zone. The opposite parental mating strategy (virgin female homozygous *Igf2*^*Flox*^ mated with male homozygous *Tpbpa*^*Cre*^) generated pregnancies with control litters. The day of the copulatory plug was defined as day 1 of pregnancy (GD1) and all procedures related to pregnant females were performed on GD16. Age-matched virgin female homozygous *Tpbpa*^*Cre*^ and female homozygous *Igf2*^*Flox*^ were used for pre-pregnancy baseline values.

For offspring studies, dams were allowed to deliver naturally and litters were standardised to six pups on postnatal day 3 (3 males and 3 females, whenever possible). Pups were weaned on day 21 and, thereafter, female and male offspring were weighed weekly. At 12 weeks of age, one animal per sex and litter received an intraperitoneal glucose or an insulin tolerance test. All offspring were euthanised at 13 weeks of age, and one animal per sex and litter underwent body composition analysis using DEXA scanning on a Lunar PIXImus densitometer (GE Lunar Corp., Madison, WI, USA). This approach was decided to maximise animal use according to the UK Home Office 3Rs directive and to avoid inflating our numbers by assessing multiple offspring from each litter.

### Diets

Two different diets were used for this study. Pregnant dams were fed *ad libitum* throughout pregnancy and lactation with a standard chow RM3 diet obtained from Special Dietary Services (Witham, UK). For the offspring studies, entire litters were randomised immediately after weaning to either the standard RM3 chow, or an obesogenic diet with a 45% kcal fat (#D12451, Research Diets, New Brunswick, NJ, USA) and 20% sucrose in tap water supplemented with AIN93G mineral and vitamin mix (MP Biomedicals, Irvine, CA, USA).

### Glucose and insulin tolerance tests

Mice were fasted for 6 hours from 08:00h on the test day. A blood sample and glucose measure was obtained from a small cut in the tail vein immediately before the intraperitoneal injection of glucose (10% weight for volume, 1 g/kg body weight) or insulin (0.75 units/kg, Actrapid; Novo Nordisk, Denmark). Blood glucose levels were quantified at 0, 15, 30, 60, 90, and 120 minutes post-injection.

### Blood collection and metabolite and hormone concentrations

Mice were anesthetised with fentanyl-fluanisone and midazolam in sterile water (10ml/g mouse, 1:1:2 ratio intraperitoneally), and blood was collected by cardiac exsanguination followed by cervical dislocation. Blood glucose was measured using a handheld glucometer (One Touch Ultra; LifeScan Inc., Malvern, PA, USA). Blood samples in EDTA-coated tubes were centrifuged at 1,500 rpm, plasma collected and stored at -20°C. Enzymatic assay kits were used to measure plasma insulin (Mercodia: 10-1247-01, Uppsala, Sweden), estradiol (Cayman Chemical: 582251, Ann Arbor, MI, USA), progesterone (Cayman Chemical: 582601), IGF2 (R&D Systems: DY792, Minneapolis, MN, USA), free (non-esterified) fatty acids (Sigma-Aldrich Corp: 11383175001, St Louis, MO, USA), cholesterol (Siemens Healthcare: DF27, Erlangen, Germany), triglycerides (Siemens Healthcare: DF69A), alanine aminotransferase (Siemens Healthcare: DF143) and alanine transaminase (Siemens Healthcare: DF41A), as per manufacturer instructions.

### Pancreatic insulin content and pancreas immunostaining

Pancreases were removed, weighed and either snap-frozen in liquid nitrogen for biochemical analysis or fixed in 4% paraformaldehyde for paraffin embedding and histological assessment. For pancreatic insulin content, whole, snap-frozen pancreas were crushed, mixed with ethanol/hydrochloric acid, and incubated overnight at -20°C. The tissue was then disrupted using a Dounce homogeniser, left overnight at 4°C and subsequently centrifuged. The supernatant was taken to measure insulin as described above. Values were expressed per mg of tissue. For insulin staining, portions of the entire pancreas were sectioned at 5µm thickness and ≥3 non-consecutive sections of each portion were stained with an antibody against insulin (1:100, Cell Signaling Technologies: 4590, Danvers, MA, USA), which was subsequently detected by biotinylated goat anti-rabbit secondary antibody (1:1000, Abcam: ab6720, Cambridge, UK), and visualised using streptavidin-conjugated peroxidase (1:500, Rockland Immunochemicals: S000-03, Gilbertsville, PA, USA) and 3,3′-Diaminobenzidine (DAB, Abcam: ab64238). Slides were counterstained with nuclear fast red (Vector Laboratories: H-3403, Burlingame, CA, USA) and sections were digitalised using a nanozoomer scanner (Hamamatsu Photonics, Shizuoka Prefecture, Japan). Analysis was performed using Image J software (National Institutes of Health, Bethesda, MD, USA) and conducted blinded to experimental groups.

### Placental IGF2 immunostaining

Paraffin-embedded placental sections cut at 5 µm were used. Antigen retrieval was performed with in 10mM sodium citrate. Slides were incubated overnight with goat anti-human IGF-II antibody (R&D Systems; AF292). Alexa Fluor® 488-AffiniPure antibody (Jackson ImmunoResearch; 705-546-147) and DAPI (Sigma-Aldrich; D9542) were used to visualize IGF2 immuno-staining and nuclei in the section, respectively. Image acquisition was performed with a LSM510 Meta confocal laser scanning microscope (Carl Zeiss, Jena, Germany) and ZEN 2009 software.

### Fetal growth and placental histological analysis

A representative number of litters were kept to assess fetal symmetry. In particular, fetuses were photographed using a Zeiss SteREO Discovery V8 microscope with an AxioCam MRc5 camera and imaged with AxioVision 4.7.2 software (Carl Zeiss AG, Oberkochen, Germany). Biometric measurements were performed using Image J software. Placentas were fixed, paraffin-embedded, exhaustively sectioned at 8µm and stained with haematoxylin and eosin to analyse junctional zone volume and endocrine cell populations using the Computer Assisted Stereological Toolbox (CAST v2.0) program, as previously described.^18^ Analyses were performed blinded to the genotype.

### Quantitative real-time PCR

Placental RNA was extracted from micro-dissected placental layers using RNeasy Plus Kits (Qiagen) following manufacture’s instruction and as previously described.^18^ Skeletal muscle RNA was extracted with RNeasy Fibrous Tissue Mini Kit (Qiagen, Hilden, Germany), adipose tissue RNA was extracted with the RNeasy Lipid Tissue Mini Kit (Qiagen) and RNA from liver, brain, gut and pancreas was obtained using an RNeasy Plus Kits (Qiagen). Following cDNA synthesis with Applied Biosystems High-Capacity cDNA Reverse Transcription Kit (Thermo Fisher Scientific, Waltham, MA, USA), quantitative real-time PCR was performed using the primers sequences specified in Table S4. Relative mRNA expression levels were then calculated using the 2^-ΔΔCT^ method.^66^ In experiments comparing expression of genes between fetal and adult tissues, due to a lack of appropriate internal control genes, an absolute quantification approach was used, with equal cDNA amounts used as template. In such cases, calculations were made using the formula 2^-ΔΔCT^, followed by arbitrary normalization to a value of 1 for the Jz.

### Placental glucose transport assay

To assess unidirectional materno-fetal clearance of the non-metabolisable radioactive tracer ^3^H-methyl D-Glucose (MeG), pregnant dams were anaesthetised with fentanyl-fluanisone (hypnorm):midazolam (hypnovel) in sterile water (1:1:2, Jansen Animal Health) and injected intravenously as previously described.^46,67^ Fetuses were then decapitated and lysed at 55°C in Biosol (National Diagnostics, Atlanta, GA USA). Fetal lysates were measured for beta emissions by liquid scintillation counting (Optiphase Hisafe II and Packard Tri-Carb, 1900; Perkin-Elmer, Waltham, MA, USA) and radioactivity (DPM; disintegrations per minute).

### Primary cell cultures of mouse placenta cells and LC-MS/MS analysis of conditioned media

All placentas within representative litters per genotype were pooled and primary placental endocrine cell cultures were performed as described previously.^68^ Briefly, placentas were enzymatically dissociated under sterile conditions and grown in a humidified atmosphere of 5% CO_2_ at 37°C. Cells were maintained with NTCT-135 medium containing 10% fetal bovine serum, 50 IU/ml ampicillin, 50 mg/ml streptomycin, and 2mM l-glutamine. Cell medium was replaced at 24h and the final length of culture was 48h. Conditioned medium from cultured placental cells was collected at 48h of culture and subjected to LC-MS/MS analysis. Of note, 24h prior to the collection of the conditioned medium, cells were washed three times with PBS and cultured in serum-free medium. The conditioned media was centrifuged for 10 min at 1000g. All LC-MS/MS experiments and data analysis has been described elsewhere.^68^ In particular, UniProt Knowledgebase (UniProtKB) gene ontology analysis (https://www.uniprot.org/) was performed to identify relevant pathways enriched in proteins uniquely present in the control or UE secretomes (165 and 125, respectively).

### LC-MS/MS analysis of mouse plasma

Extraction and analysis of circulating proteins in plasma was performed as described elsewhere.^68^ Briefly, mouse plasma was thawed and 6M guanidine hydrochloride was added in 1:1 proportion. Precipitation of proteins was performed by adding 75% acetonitrile in water at a ratio of 1:6 (diluted plasma:acetonitrile) followed by sample centrifugation at 2900 × *g* for 10 min at 4°C. Extracts were acidified and analysed using nano LC-MS/MS and the data searched using PEAKS. Peptides from specific proteins of interest in the PEAKS search result (80 proteins in total) were selected for targeted quantitation in the raw LC-MS/MS files. The peak area of each peptide was expressed as a ratio of a digested peptide from the spiked internal standard peptide (bovine insulin). Functional analysis was performed using DAVID (Database for Annotation, Visualization and Integrated Discovery; v6.8).

### Western immunoblotting

Protein was extracted from livers and placental Jz using RIPA buffer (Sigma-Aldrich Corp., R0278). Protein concentration was determined by a Pierce™ BCA Protein Assay Kit (Thermo Fisher Scientific). After electrophoresis and protein transfer onto 0.2µm nitrocellulose membranes (Bio-Rad Laboratories Inc., Hercules, CA, USA), membranes were blocked in fetal bovine serum or skimmed milk and incubated with primary antibodies (Table S5) overnight on a rocker at 4°C. Following washes, membranes were incubated with secondary antibody (dilution 1:10,000; NA931 or NA934; Amersham ECL Mouse/Rabbit IgG, HRP-linked). The iBright 1500 Imaging System (Invitrogen) was used to detect immunocomplexes in the membrane after application of SuperSignal™ West Femto Maximum Sensitivity Substrate (Thermo Fisher Scientific). Image J software was used to quantitate the densitometric intensity of immunoreactive bands. To control for protein loading, membranes were stained and normalised with Ponceau S.^69^

### RNA sequencing methods

### Nucleus dissociation

Samples were pooled together to yield 2 control samples and 2 UE samples (4 placenta per sample; offspring sex 2M:2F per sample). Samples were stored at -80°C until dissociation. Nucleus dissociation was performed as previously described.^70^ Samples were homogenised separately using a Dounce homogeniser (10 strokes with pestle A and 10 strokes with pestle B) containing 1 ml of homogenisation buffer (100 µM of DTT [Sigma–Aldrich], 0.1% Triton X-100 [Sigma–Aldrich], 2X EDTA protease inhibitor [Roche, Basel, Switzerland], 0.4U/ul of protector RNase inhibitor [Roche, 40U/ul] in nuclei isolation medium [250 mM of sucrose, 25 mM of KCl (Ambion), 5 mM of MgCl2 (Ambion), and 10 mM of Tris buffer pH 8.0 (Ambion) in nuclease-free water (Ambion)] with 1 ml/ ml of DRAQ5 [Biostatus]) on ice. Once dissociated, homogenates were centrifuged for 10 minutes at 900 x *g* at 4°C. The supernatant was removed, and each pellet was resuspended in 900ul of homogenisation buffer with 25% Optiprep (diluted in iodixanol dilution medium [250mM sucrose, 150mM KCl, 30mM MgCl_2_, and 60mM Tris buffer pH 8.0 in nuclease-free water]). Each suspension was carefully layered on top of separate 900ul of 29% OptiPrep solutions (diluted in iodixanol dilution medium), and samples were centrifuged at 13,500 x *g* for 20 minutes at 4°C. Each nuclei pellet was removed and resuspended in 1ml of wash buffer (1% BSA and 0.4U/ ul protector RNase inhibitor in PBS) before being passed through a 40um cell strainer into a polypropylene tube.

### Fluorescence-activated nucleus sorting

Nucleus sorting was performed using a BD high-speed influx cell sorter (BD Biosciences, Franklin Lakes, NJ, USA). Forward scatter (FSC, size), side scatter (SSC, granularity) and DRAQ5 (anthraquinone dye with high affinity for double-stranded DNA) fluorescence nuclear staining were used to capture singlets and filter out debris. DRAQ5 fluorescence was detected at 647/670nm. A total of 15,000 particles from each sample were sorted into separate tubes for single nuclear RNA-sequencing. Nucleus sorting was performed by the Flow Cytometry Core Facility at the Cambridge Institute for Medical Research.

### Single nucleus RNA-sequencing (snSeq)

Sequencing libraries for the 4 samples (2 control and 2 UE samples) were generated using the 10X genomics chromium single-cell 3’ reagent kits (version 3.1). Nuclei suspensions with master mix were loaded onto a chromium next GEM chip with gel beads and partitioning oil to generate GEMs containing a single nucleus. RNA from lysed nuclei in the GEMs was then reversed transcribed and cDNA was amplified for 19 cycles. The amplified cDNA was used to generate the barcoded 3’ library according to the 10X genomics protocols, and paired-end sequencing was performed using an Illumina NovaSeq 6000 (San Diego, CA, USA; Read 1: 28bp Read 2: 91 bp). Library preparation and sequencing were performed by the IMS Genomics and Transcriptomic Core and the Cancer Research UK Cambridge Institute Genomics Core.

### Raw data processing and downstream analysis

Raw sequencing reads were aligned to the mouse genome (mm10) with introns included during alignment using CellRanger package (10X Genomics, version 6.1.1). Ambient RNA was removed from each nucleus using CellBender/ CellRanger raw filter matrix as an input.^63^ Downstream analysis was then performed on the filtered count matrix from the CellBender output using Seurat version 4.1.0.^64^

Nuclei expressing less than 500 genes or 1000 transcripts were identified as low quality nuclei and removed. Nuclei expressing more than 10% mitochondrial RNA or more than a total of 40,000 reads were also excluded from analysis. Doublets were identified using scDblFinder v1.9.12^65^ and removed from the datasets. Each dataset was normalised using sctransform^71^ and then integrated using 3000 features. PCA was carried out on the integrated dataset, and the top 25 principal components were used for UMAP dimensional reduction. Cluster analysis was performed using the Louvain algorithm.^72^ A cluster which showed expression of multiple cell type markers was removed. Normalization (sctransform), integration (3000 features), and clustering was re-performed on this subset of the data.

Maternal clusters were identified through absence of Y-linked *Ddx3y*, and expression of this transcript in fetal cells was used to infer sex of the offspring. Cell types of each cluster were identified based on expression of canonical marker genes (spongiotrophoblasts = *Prl8a9*, glycogen cells = *Igfbp7*, decidual stroma = *Pgr*, endothelial cells = *Pecam1*, immune cells = Ptprc, fetal mesenchyme = *Gata4, Wnt5b*, SynTI = *Stra6*, sinusoidal trophoblast giant cells = Ctsq, SynTII = Gcgr, labyrinthine trophoblast = *Cdh1*).^37^ Cluster markers were calculated using the ‘FindAllMarkers’ Seurat function, which utilises the Wilcoxon Rank Sum test to identify differentially expressed genes between clusters. The top two genes with a logFC greater than 1.5, expressed in at least 80% of cells in the cluster and expressed in less than 20% of the rest of the dataset were used to name each cluster. Junctional zone cells were identified based on clusters showing high expression of *Tpbpa*, and expression of spongiotrophoblast markers or glycogen cell markers and a subset was created for further analysis.

Differential expression of genes between the control and UE groups was calculated using a pseudo-bulked matrix, where each sample was further split based on inferred sex and transcript counts per cluster were summed for each sample. DESeq2^73^ differential expression analysis was performed on the pseudo-bulked matrix. Genes with a fold change greater than 2 between control and UE samples and an adjusted p value lower than 0.05 were classified as DEGs (differentially expressed genes). DAVID analysis, followed by REVIGO^62^ filtering of redundant gene ontology (GO terms) were used to identify biological processes enriched in DEGs within each Jz cell-type cluster.

### Statistical analysis

All data are shown with individual data points and the mean±SEM where possible. Feto-placental weights and placental transport assays were analysed by ANOVA with repeated measures (MIXED model) using the SAS/STAT Software (Statistical System Institute Inc. Cary, NC, USA). Each mother was considered the subject, the genotype the main factor and litter size was added as covariate. Offspring biometry data were analysed using litter means by two-way ANOVA. Glucose and insulin tolerance tests were analysed by repeated measures ANOVA followed by Tukey post-hoc tests for mean comparisons. Maternal data were analysed by ANOVA using GraphPad Prism software (GraphPad Software Inc., La Jolla, CA, USA). For analyses only using two groups (namely litter size, steroid concentrations, and western blotting data), the Shapiro-Wilk test was performed to determine if data followed the normal distribution and then statistical analysis was performed using Mann-Whitney or unpaired Student’s t-tests as appropriate. The numbers of samples used for each experiment are shown within the respective figure or figure legends. A value of p<0.05 was considered statistically significant.

## Supplementary Material

Supplemental information can be found online at https://doi.org/10.1016/j.cmet.2023.06.007.

Supplemental information

Supplemental information 1

Supplemental information 2

Supplemental information 3

Supplemental information 4

## Data Availability

The authors declare that the data generated and analysed to support the findings of this study are available within the paper and its supplementary files, or available from the authors upon reasonable request. Single-nuclear RNA sequencing data are available from NCBI Gene Expression Omnibus (GEO) repository accession number GEO: GSE229514. Source data are provided in Data S1. This manuscript did not generate any code.

## Highlights

- Placenta endocrine cell (Jz) IGF2 loss impairs maternal adaptation in pregnant mice
- Jz IGF2 loss reduces maternal glucose and lipid availability for fetal growth
- IGF2 regulates metabolism, protein synthesis, and hormone secretion of Jz cells
- Lack of Jz IGF2 programs offspring for postnatal metabolic dysfunction
