# Supplementary material for: Fetal manipulation of maternal metabolism is a critical function of the imprinted Igf2 gene
Source: Cell Metab. Author manuscript; Available in PMC 2026 Aug 29. (PMC7619417; doi:10.1016/j.cmet.2023.06.007)
Supplement: Supplementary file 1 [file EMS217968-supplement-Supplemental_information.pdf]

Cell Metabolism, Volume *35*

Supplemental information

Fetal manipulation of maternal metabolism

is a critical function of the imprinted *Igf2* gene

Jorge Lopez-Tello, Hannah E.J. Yong, Ionel Sandovici, Georgina K.C. Dowsett, Efthimia R. Christoforou, Esteban Salazar-Petres, Rebecca Boyland, Tina Napso, Giles S.H. Yeo, Brian Y.H. Lam, Miguel Constancia, and Amanda N. Sferruzzi-Perri

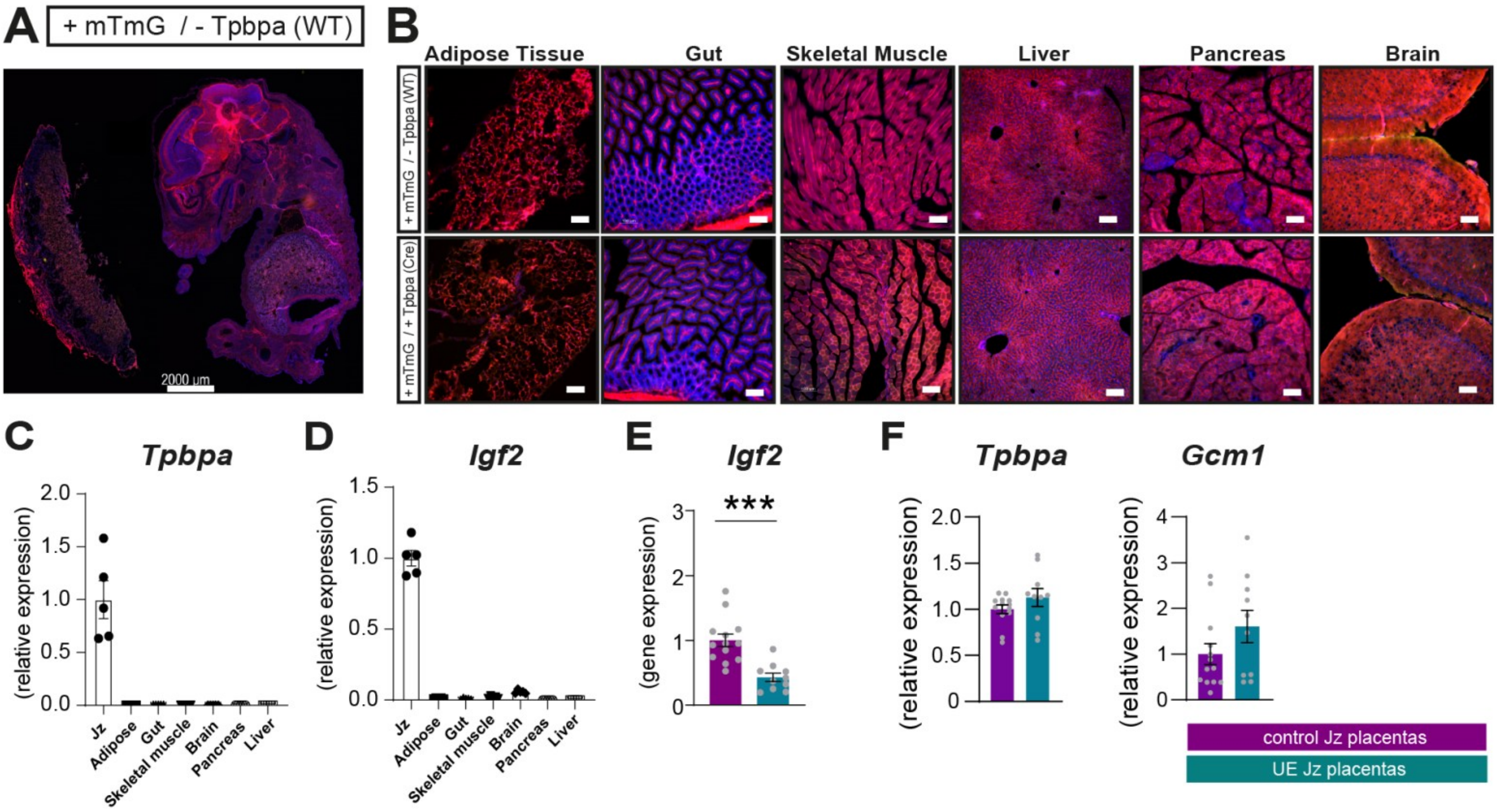

Supplementary Figure 1. *Tpbpa* and *Igf2* expression levels. Related to Figure 1.

(A) Negative control litter mate for mT/mG x *Tpbpa*^{Cre} validation experiment shown in Figure 1A. The conceptus was harvested on day 16 of gestation.

(B) Adult tissues (>5 weeks of age) from animals that did or did not carry the *Tpbpa*^{Cre}. Both groups were mTmG positive (scale bar is 100µm).

(C-D) Expression levels for *Tpbpa* and *Igf2* in dissected placental junctional zone (Jz; on gestational day 16) and adult tissues (5 weeks of age) showing that *Tpbpa* and *Igf2* (arbitrarily normalised to 1) are much higher in the Jz compared to the other tissues/organs (n=5/group).

(E-F) Relative mRNA expression levels of *Igf2*, *Tpbpa* (junctional zone marker) and *Gcm1* (labyrinth zone marker) in dissected junctional zone samples (levels in control samples are arbitrarily normalised to 1). Note that Jz samples have some labyrinth zone contamination (n=10-13 placentas/group). Analysis performed by Student t test or Mann-Whitney test. ***p<0.001.

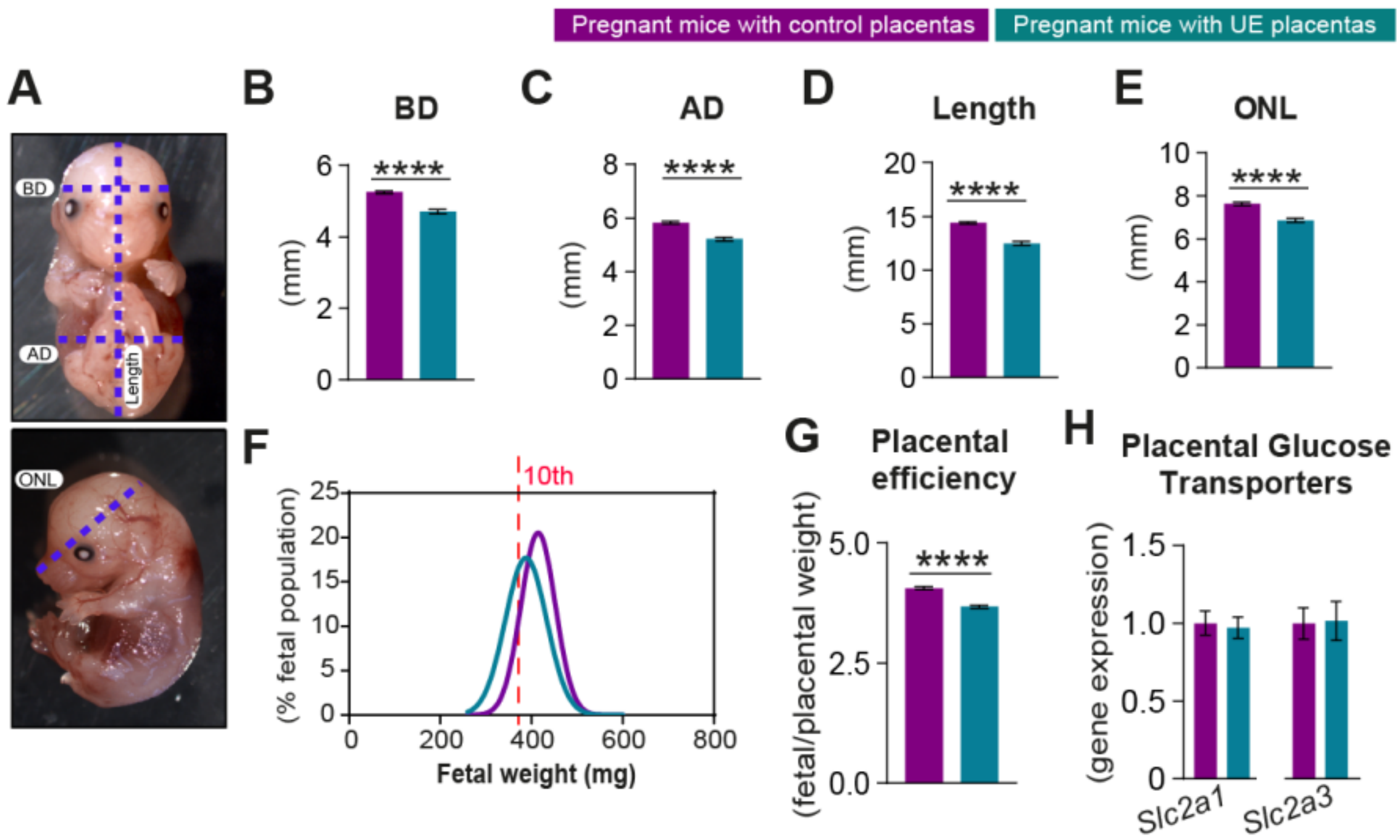

Supplementary Figure 2. Additional feto-placental parameters on gestational day 16. Related to Figure 1.

(A) Representative image showing measured fetal growth parameters of biparietal diameter

(BD), abdominal diameter (AD), length, and occipital to nose length (ONL).

(B-E) Measurements of fetal growth parameters (n=6 litters/group).

(F) Distribution of control and UE fetal weights with a dotted line showing the 10th centile cut-off (372mg, representing 10th centile of control fetuses) used to define growth-restricted fetuses.

(G) Crude placental efficiency assessment, defined as the ratio between the fetal and placental weights (n=28-32 litters/group). Note that this parameter does not reflect an intrinsic placental defect, as it can be modified by fetal demand for nutrients, placental transport capacity or maternal nutrient availability.

(H) Relative mRNA expression of placental glucose transporters (n=14 fetuses/6 litters/group).

Data is presented as mean±SEM. Data were analysed by ANOVA tests with repeated measures (MIXED model), considering the mother as the subject, the genotype as the main factor, and the litter size as a covariate. ****$p<0.0001$.

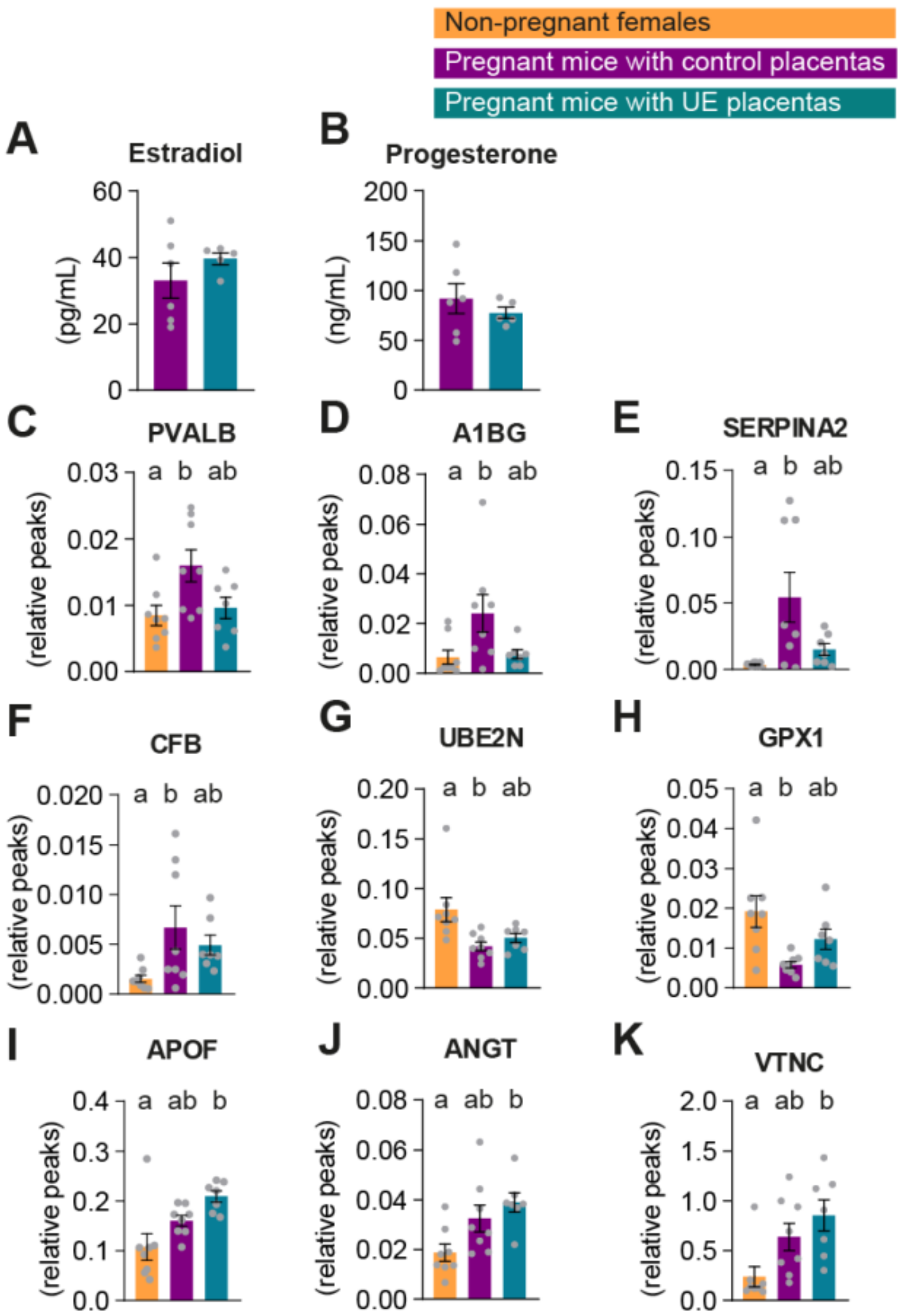

Supplementary Figure 3. Measurements of circulating steroid hormones and proteins in maternal blood at day 16 of pregnancy and non-pregnant females. Related to Figure 3.

(A-B) Circulating concentrations of steroid hormones, estradiol and progesterone as measured by ELISA (n=5-6/group).

(C-K) Relative plasma levels of proteins identified by LC-MS/MS in the circulation of UE dams that were intermediate between non-pregnant and pregnant state (C-H) or significantly different to non-pregnant state (I-K) (n=7-8/group).

Data is presented as mean±SEM, with individual data points shown. Student t-tests for two groups analyses or one-way ANOVA with Tukey post-hoc tests for mean comparisons were performed, as appropriate. Different letters (a, b) indicate significant differences between groups by one-way ANOVA ($p<0.05$).

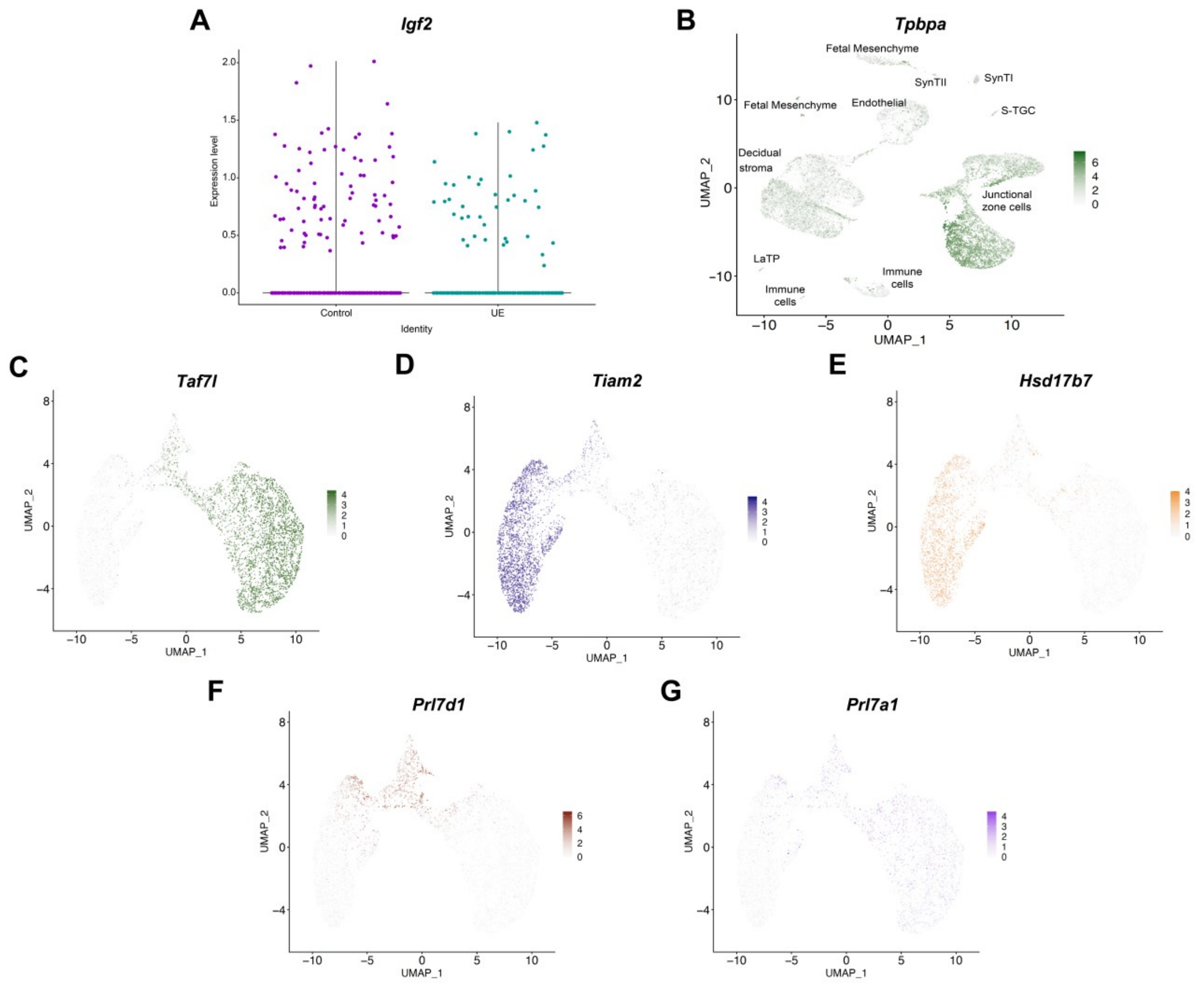

Supplementary Figure 4. Additional information regarding the single nuclear RNA-sequencing. Related to Figure 5.

(A) A violin plot showing the overall expression levels of *Igf2* in all placental endocrine cells in control vs UE.

(B) A UMAP plot showing the expression of *Tpbpa* in all cell populations identified via single-nuclear RNA sequencing (Jz, junctional zone).

(C-G) UMAP plots showing the expression of top marker genes for each cluster within the Jz.

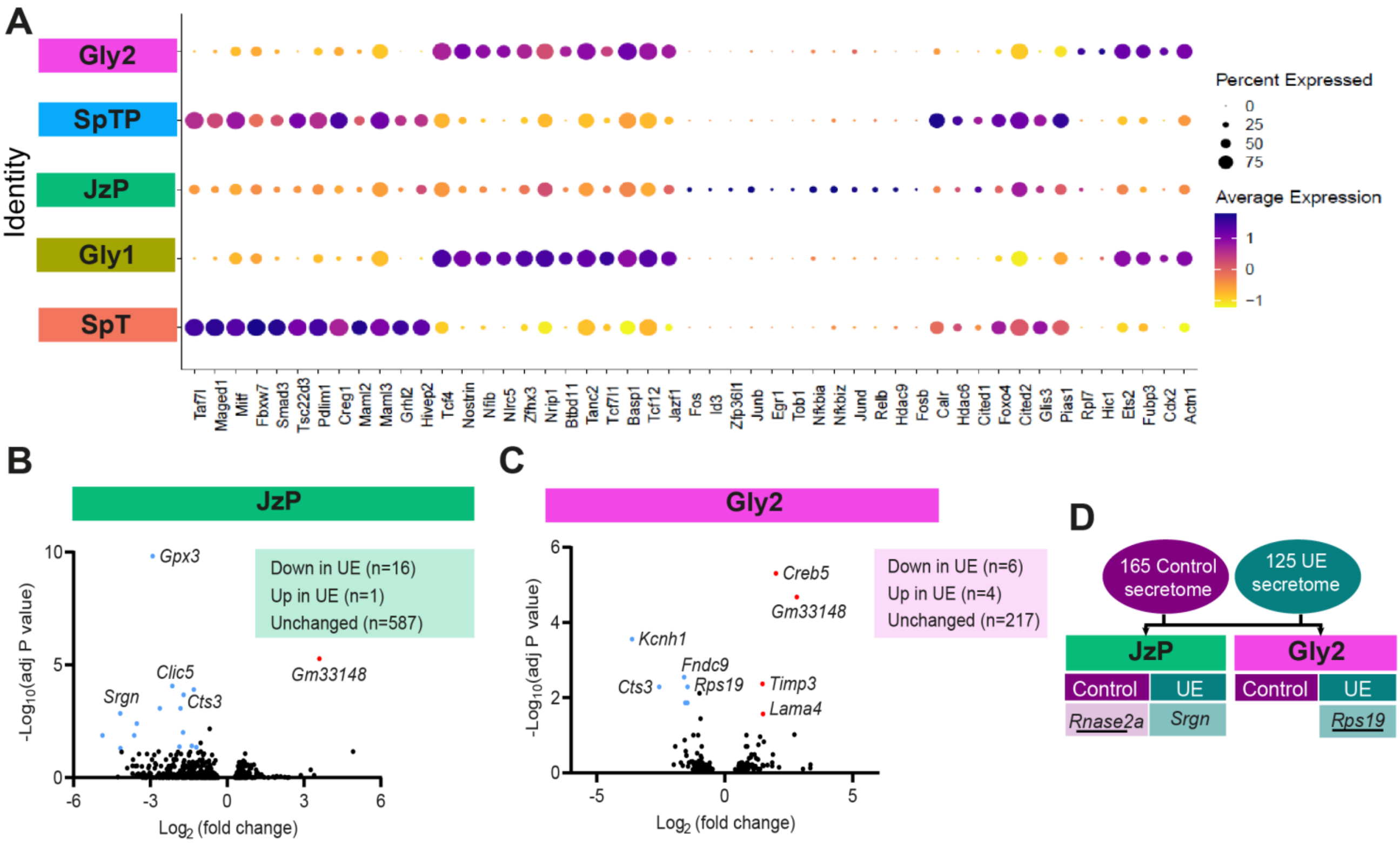

Supplementary Figure 5. Further information about the single cell nuclear RNA-sequencing and pathways analysis. Related to Figure 5.

(A) Dot plot showing 49 transcription factors uniquely expressed in placental endocrine cell clusters. These were based on the top 12 transcription factors identified as markers for each of the 5 Jz cell clusters that were uniquely expressed by Jz cells. The intensity represents the scaled expression level and the size represents the percent of cells expressing the gene. Genes are listed on the x-axis and clusters on the y-axis.

(B-C) Volcano plot representation of DEGs in JzP and Gly2 populations.

(B-C) Volcano plot representation of differentially expressed genes (DEGs) in JzP (junctional zone precursors) and Gly2 (glycogen type 2) cell populations.

(D) Overlapping of the datasets generated from the placental secretome (proteins that were detected in one group, but not the other: 125 unique proteins in UE, and 165 unique proteins in controls) and the DEGs detected by snRNA-seq. Genes underlined are found in more than one cell cluster.

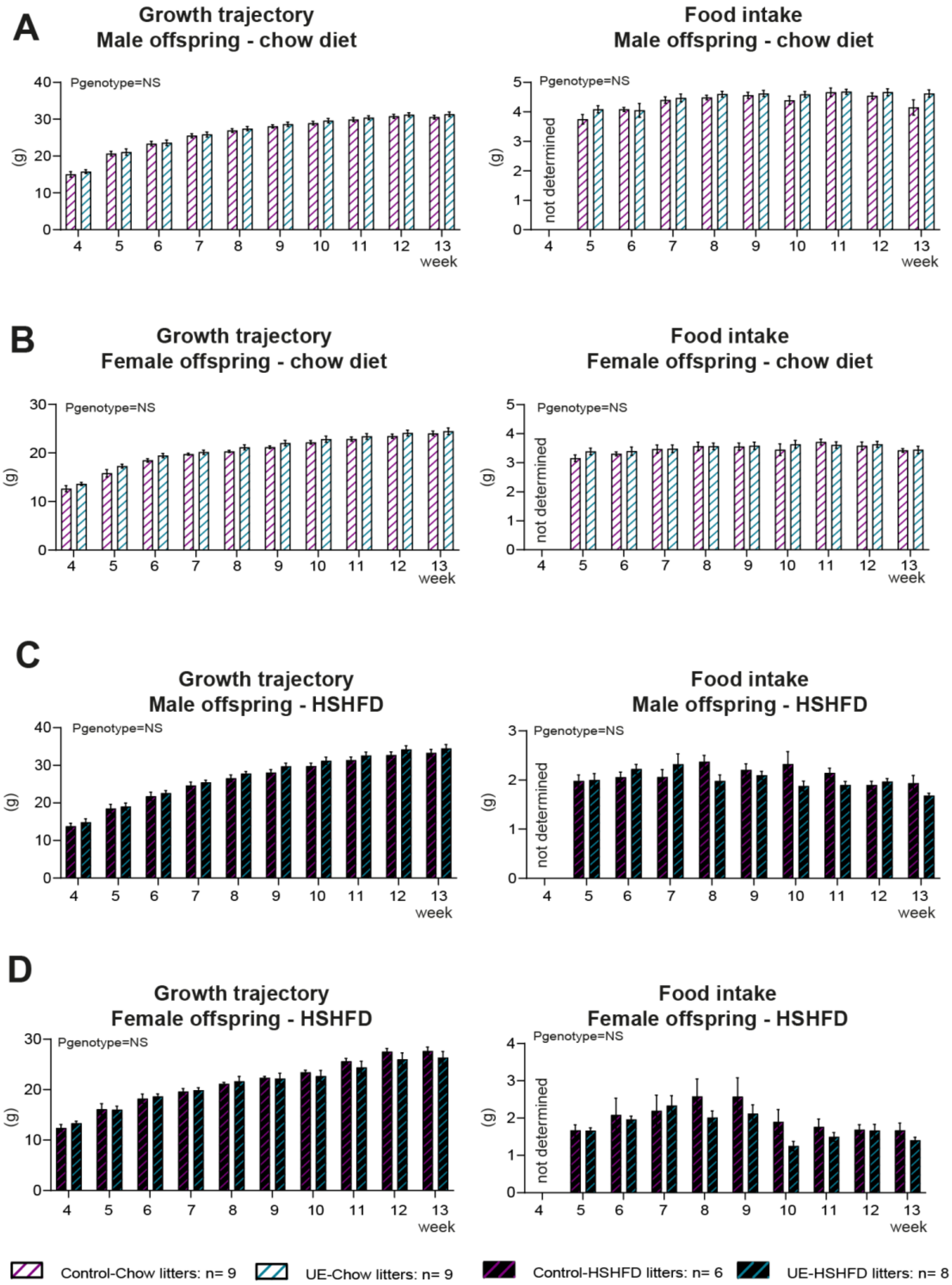

Supplementary Figure 6. Growth trajectories and food intake of male and female offspring. Related to Figure 6.

(A-D) Weekly measures of weight and food intake of male and female offspring supported by control and UE placentas from 4 weeks and 5 weeks of age respectively. (n=6-9/group)

Data is presented as mean±SEM and analysed using two-way ANOVA repeated measures with Bonferroni post hoc tests for mean comparisons.

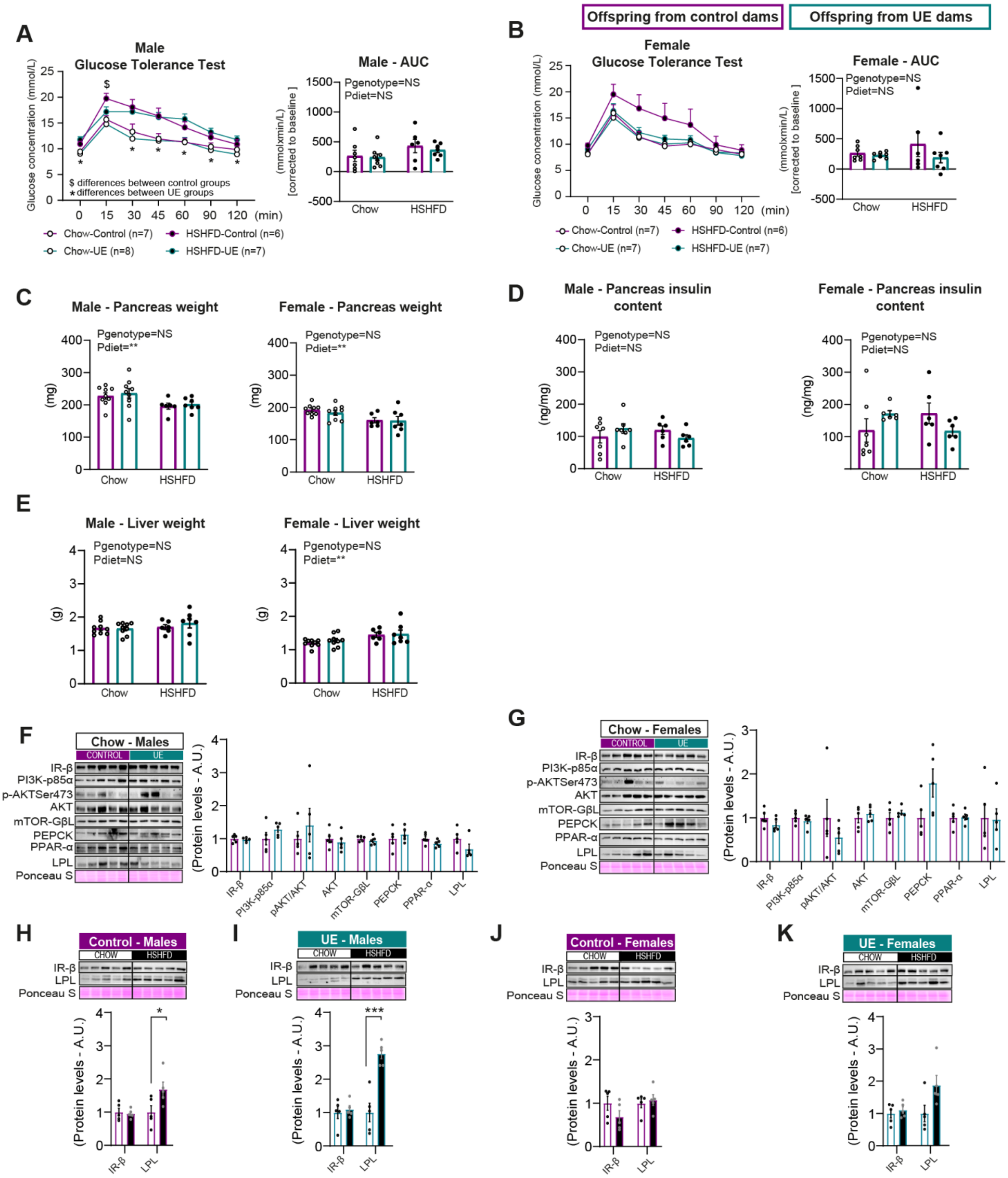

Offspring from control dams
Offspring from UE dams
A
Male
Glucose Tolerance Test
Glucose concentration (mmol/L)
$ differences between control groups
* differences between UE groups
(min)
Chow-Control (n=7)
HSHFD-Control (n=6)
Chow-UE (n=8)
HSHFD-UE (n=7)
Male - AUC
Pgenotype=NS
Pdiet=NS
(mmolxmin/L)
[corrected to baseline]
Chow
HSHFD
B
Female
Glucose Tolerance Test
Glucose concentration (mmol/L)
(min)
Chow-Control (n=7)
HSHFD-Control (n=6)
Chow-UE (n=7)
HSHFD-UE (n=7)
Female - AUC
Pgenotype=NS
Pdiet=NS
C
Male - Pancreas weight
Pgenotype=NS
Pdiet=**
(mg)
Female - Pancreas weight
Pgenotype=NS
Pdiet=**
D
Male - Pancreas insulin content
Pgenotype=NS
Pdiet=NS
(ng/mg)
Female - Pancreas insulin content
Pgenotype=NS
Pdiet=NS
E
Male - Liver weight
Pgenotype=NS
Pdiet=NS
(g)
Female - Liver weight
Pgenotype=NS
Pdiet=**
F
Chow - Males
CONTROL
UE
IR-β
PI3K-p85α
p-AKTSer473
AKT
mTOR-GβL
PEPCK
PPAR-α
LPL
Ponceau S
(Protein levels - A.U.)
pAKT/AKT
G
Chow - Females
H
Control - Males
CHOW
HSHFD
I
UE - Males
J
Control - Females
K
UE - Females

Supplementary Figure 7. Glucose tolerance tests, pancreas analyses and measurements of hepatic proteins involved in the control of glucose and lipids, in male and female offspring on chow and high sugar high fat diet (HSHFD). Related to Figure 6 and 7.

(A-B) Intraperitoneal glucose tolerance tests were performed at 12 weeks of age. Graphs on the right show area under glucose curve (AUC) (n=6-8/group/sex; each dot represents a different litter).

(C-D) Pancreas weight (n=6-9/group/sex; each dot represents a different litter) and pancreatic insulin content at 13 weeks of age (n=6-7/group/sex).

(E) Absolute liver weights at 13 weeks of age (n=6-9/group/sex).

(F-G) Immunoblots with a representative Ponceau staining image and relative levels of proteins involved in metabolism in liver from male and female offspring on a chow diet at 13 weeks of age (n=5/group/sex; each dot represents a different litter).

(H-K) Immunoblots and a representative Ponceau staining as loading control and relative levels of proteins involved in metabolism in liver from male and female offspring on a HSHFD at 13 weeks of age (n=5/group/sex; each dot represents a different litter).

Data are presented as mean±SEM with individual data points shown. Two-way ANOVAs were performed, with Tukey post-hoc tests for mean comparisons. Western blots were analysed by Student t-test (*$p<0.05$). Different symbols (*, $) indicate significant differences between groups by two-way ANOVA with repeated measures.

Table S4. List of primers used for qPCR. Related to Figure S1, S2 and STAR Methods.

Gene	Forward	Reverse
Igf2	CTTGTTGACACGCTTCAGTTTG	GGGTGGCACAGTATGTCTCC
Tpbpa	TGAAGAGCTGAACCACTGGA	CTTGCAGTTCAGCATCCAAC
Gcm1	TTTCCAGTCCAAAGGCGAGC	TGACTCGGGATTTCAGCAGG
Gapdh	GGGAAATGAGAGAGGCCCAG	GAACAGGGAGGAGCAGAGAG
Ywhaz	AAACAGCTTTCGATGAAGCCA	CATCTCCTTGGGTATCCGATGT
Hrpt	CAGGCCAGACTTTGTTGGAT	TTGCGCTCATCTTAGGCTTT
Slc2a1	GCTTATGGGCTTCTCCAAACT	GGTGACACCTCTCCCACATAC
Slc2a3	GATCGGCTCTTTCCAGTTTG	CAATCATGCCACCAACAGAG
Ubc	GGAGTCGCCCGAGGTCA	AAAGATCTGCATCGTCTCTCTCAC

Table S5. List of antibodies used for western blotting. Related to Figures 2, 4, 5, 7, S7 and STAR Methods.

Antibody	Catalogue number	Dilution
Insulin receptor beta	Cell Signalling, 3020	1:1000
PI3K-p85 alpha	Millipore, 06-195	1:500
Phospho-Akt (Ser473)	Cell Signalling, 4060	1:1000
AKT	Cell Signalling, 9272	1:1000
GβL (86B8)	Cell Signalling, 3274	1:1000
PEPCK	Santa Cruz, sc-271029	1:200
Lipoprotein lipase	Abcam, ab21356	1:1000
PPAR alpha	Abcam, ab3484	1:1000
PPAR gamma	Santa Cruz, sc-7273	1:1000
Phospho-AMPK (Thr172)	Cell Signalling, 2535	1:1000
AMPK	Cell Signalling, 5832	1:1000
STAT3	Millipore, 07-2173	1:1000
STAT5	Cell Signalling, 25656	1:1000
S6 Ribosomal protein	Cell Signalling, 3944	1:1000
IGF-II Receptor	Cell Signalling, 15128	1:1000
IGF-I Receptor	Cell Signalling, 9750	1:1000
