# Supplementary material for: Fetal manipulation of maternal metabolism is a critical function of the imprinted Igf2 gene
Source: Cell Metab. Author manuscript; Available in PMC 2026 Aug 29. (PMC7619417; doi:10.1016/j.cmet.2023.06.007)
Supplement: Supplementary file 2 [file EMS217968-supplement-Supplemental_information_4.zip › Data S1.pdf]

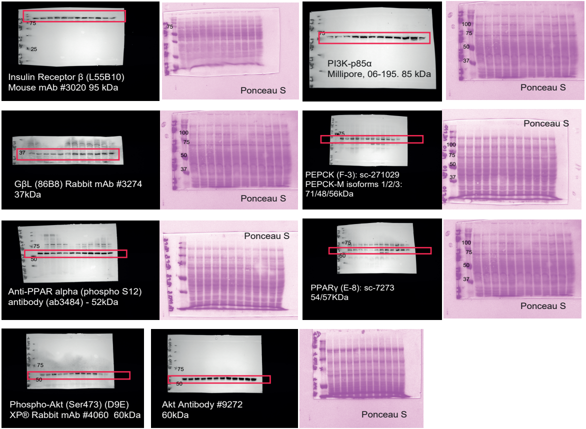


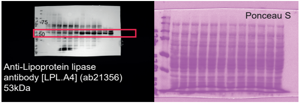


# Figure 2
# Maternal liver western blots

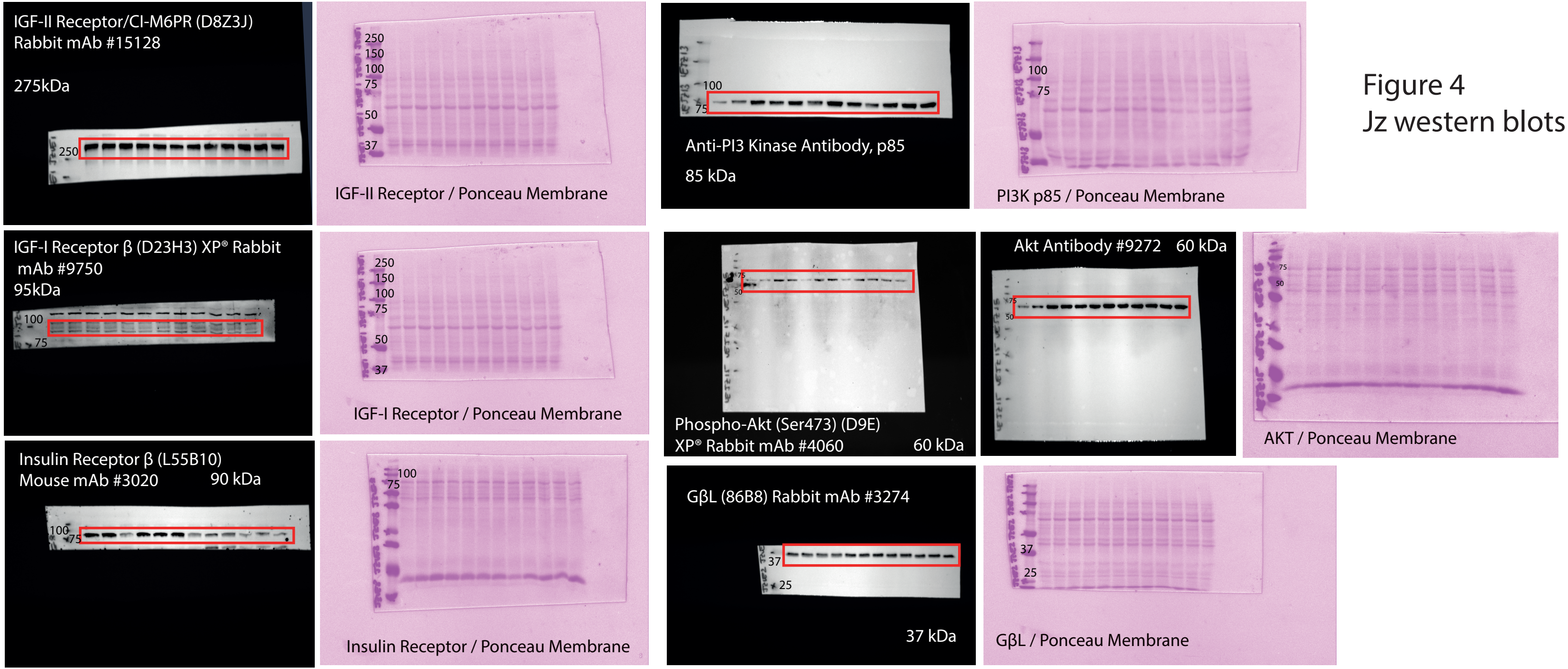


Figure 4
Jz western blots

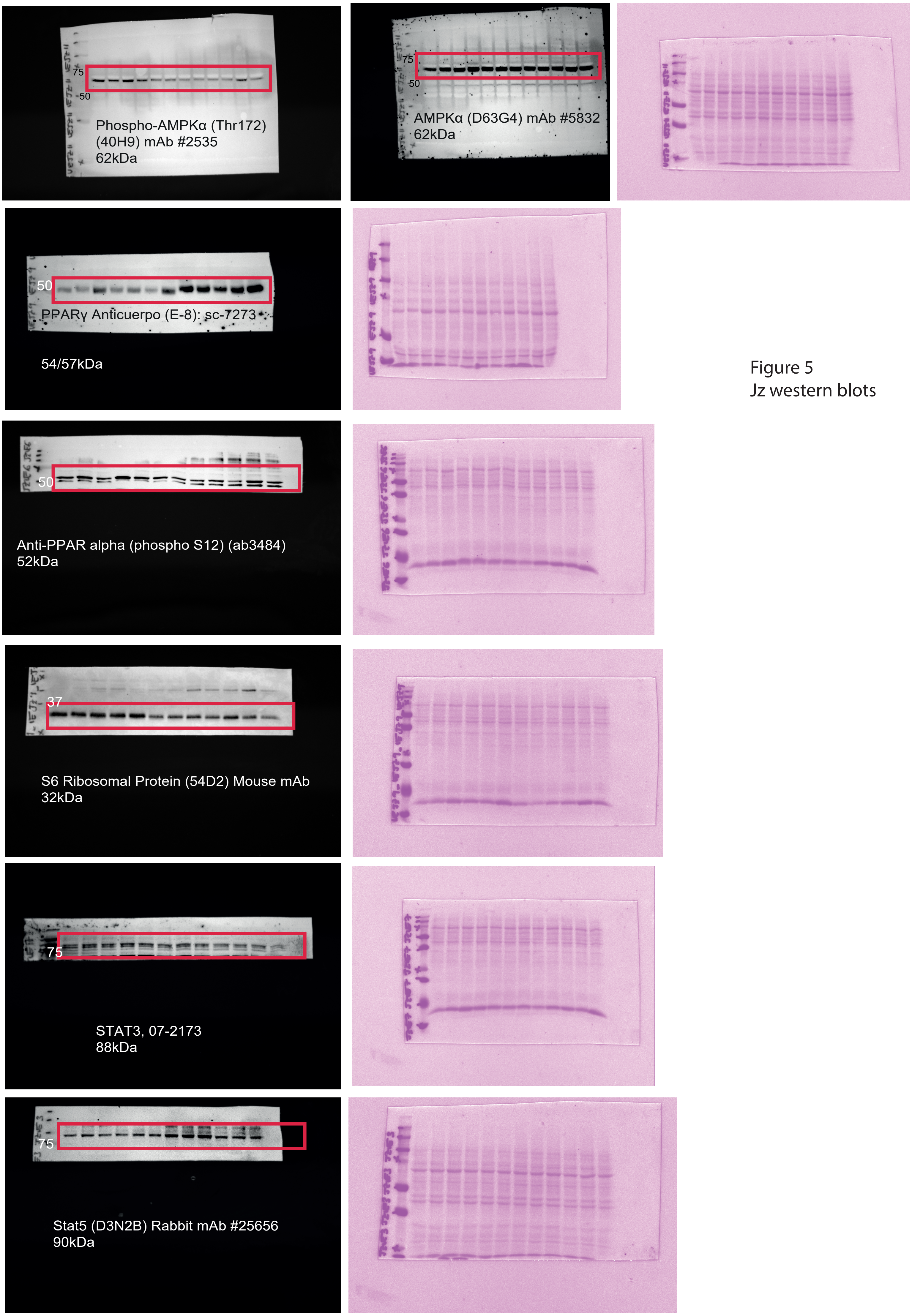


Figure 5
Jz western blots

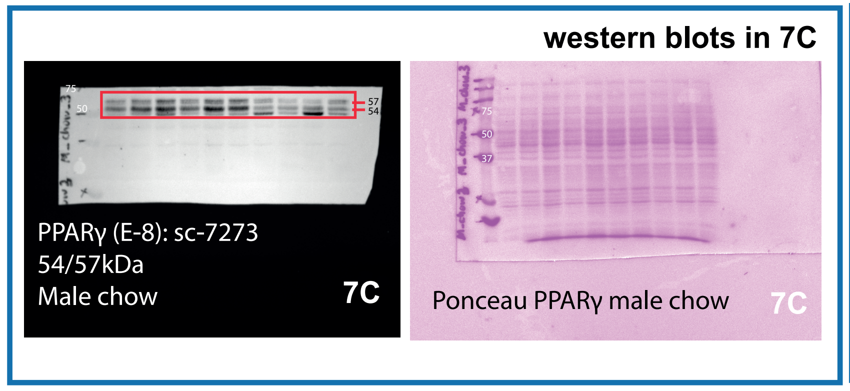
western blots in 7C
PPARγ (E-8): sc-7273
54/57kDa
Male chow
7C
Ponceau PPARγ male chow
7C

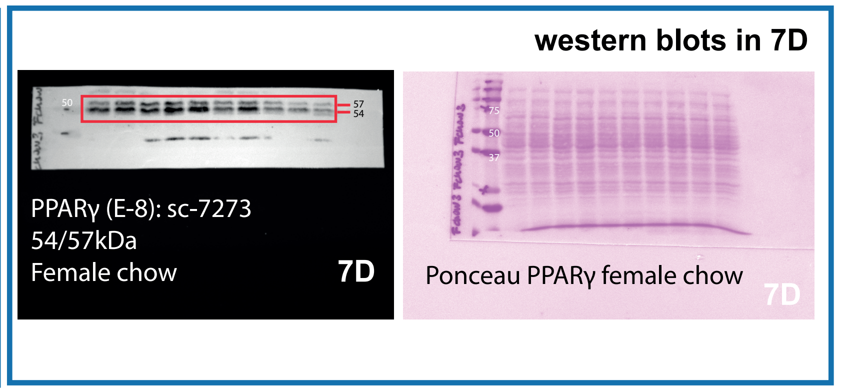
western blots in 7D
PPARγ (E-8): sc-7273
54/57kDa
Female chow
7D
Ponceau PPARγ female chow
7D

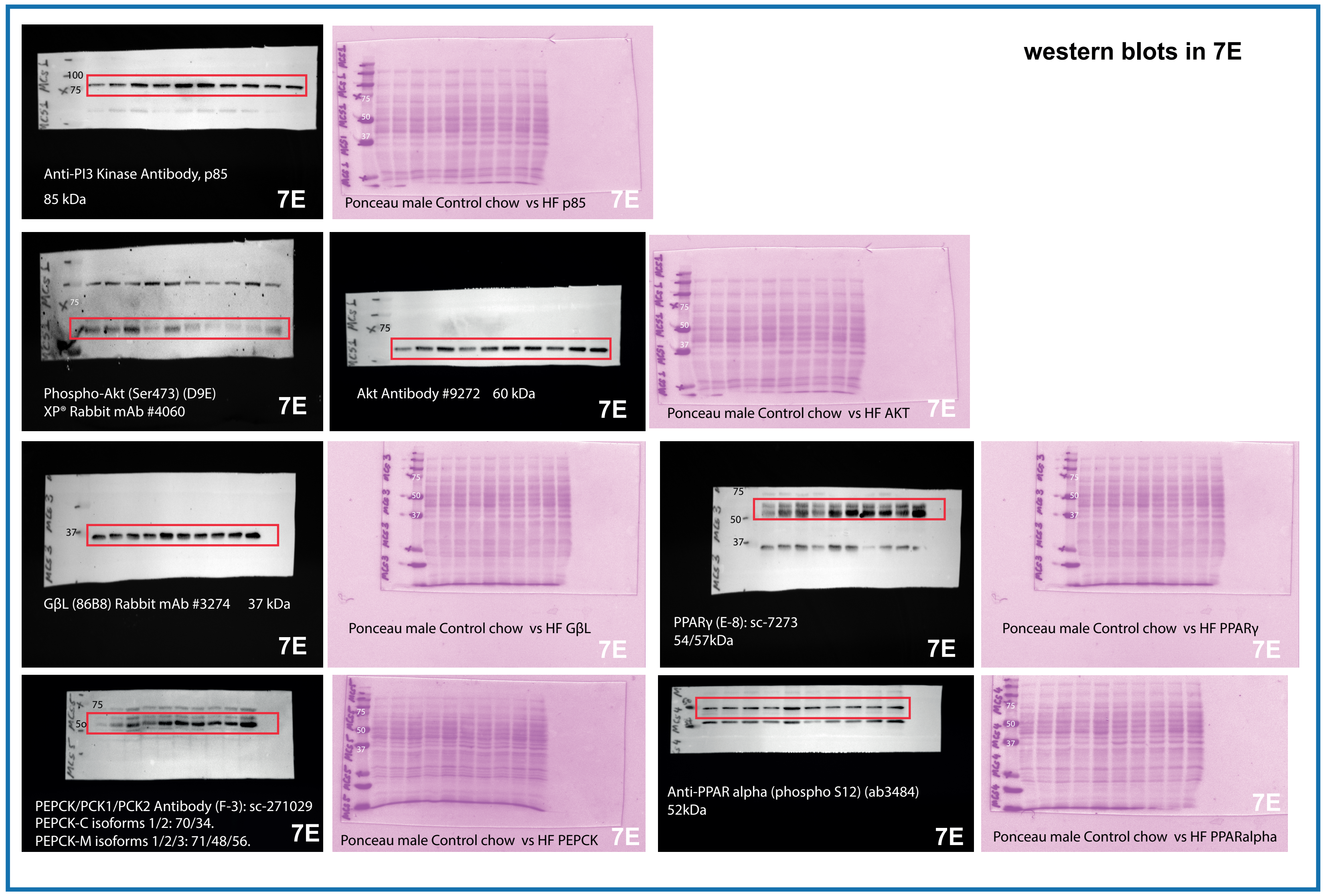
western blots in 7E
Anti-PI3 Kinase Antibody, p85
85 kDa
7E
Ponceau male Control chow vs HF p85
7E
Phospho-Akt (Ser473) (D9E)
XP® Rabbit mAb #4060
7E
Akt Antibody #9272 60 kDa
7E
Ponceau male Control chow vs HF AKT
7E
GβL (86B8) Rabbit mAb #3274 37 kDa
7E
Ponceau male Control chow vs HF GβL
7E
PPARγ (E-8): sc-7273
54/57kDa
7E
Ponceau male Control chow vs HF PPARγ
7E
PEPCK/PCK1/PCK2 Antibody (F-3): sc-271029
PEPCK-C isoforms 1/2: 70/34.
PEPCK-M isoforms 1/2/3: 71/48/56.
7E
Ponceau male Control chow vs HF PEPCK
7E
Anti-PPAR alpha (phospho S12) (ab3484)
52kDa
7E
Ponceau male Control chow vs HF PPARalpha
7E

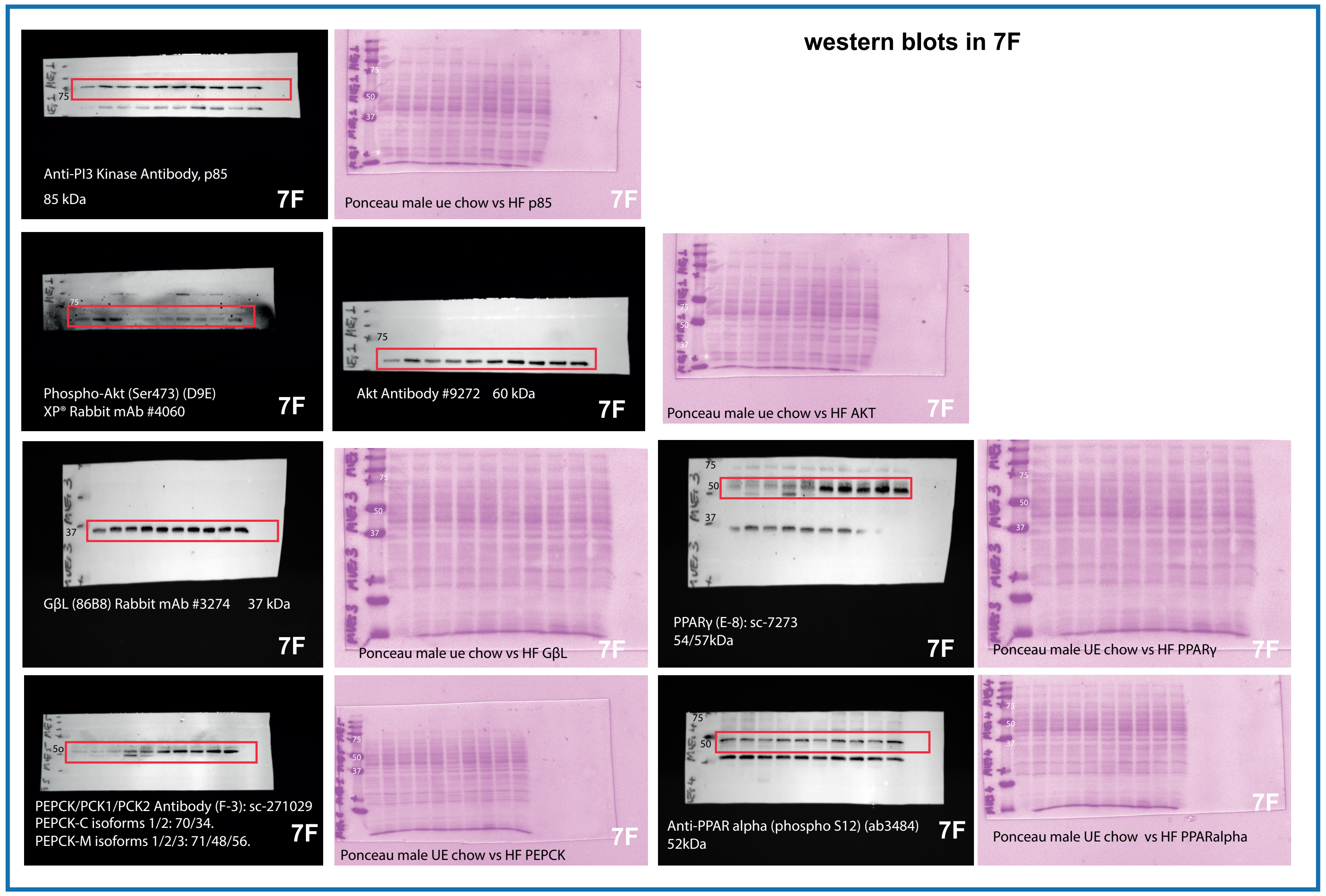
western blots in 7F
Anti-PI3 Kinase Antibody, p85
85 kDa
7F
Ponceau male ue chow vs HF p85
7F
Phospho-Akt (Ser473) (D9E)
XP® Rabbit mAb #4060
7F
Akt Antibody #9272 60 kDa
7F
Ponceau male ue chow vs HF AKT
7F
GβL (86B8) Rabbit mAb #3274 37 kDa
7F
Ponceau male ue chow vs HF GβL
7F
PPARγ (E-8): sc-7273
54/57kDa
7F
Ponceau male UE chow vs HF PPARγ
7F
PEPCK/PCK1/PCK2 Antibody (F-3): sc-271029
PEPCK-C isoforms 1/2: 70/34.
PEPCK-M isoforms 1/2/3: 71/48/56.
7F
Ponceau male UE chow vs HF PEPCK
7F
Anti-PPAR alpha (phospho S12) (ab3484)
52kDa
7F
Ponceau male UE chow vs HF PPARalpha
7F

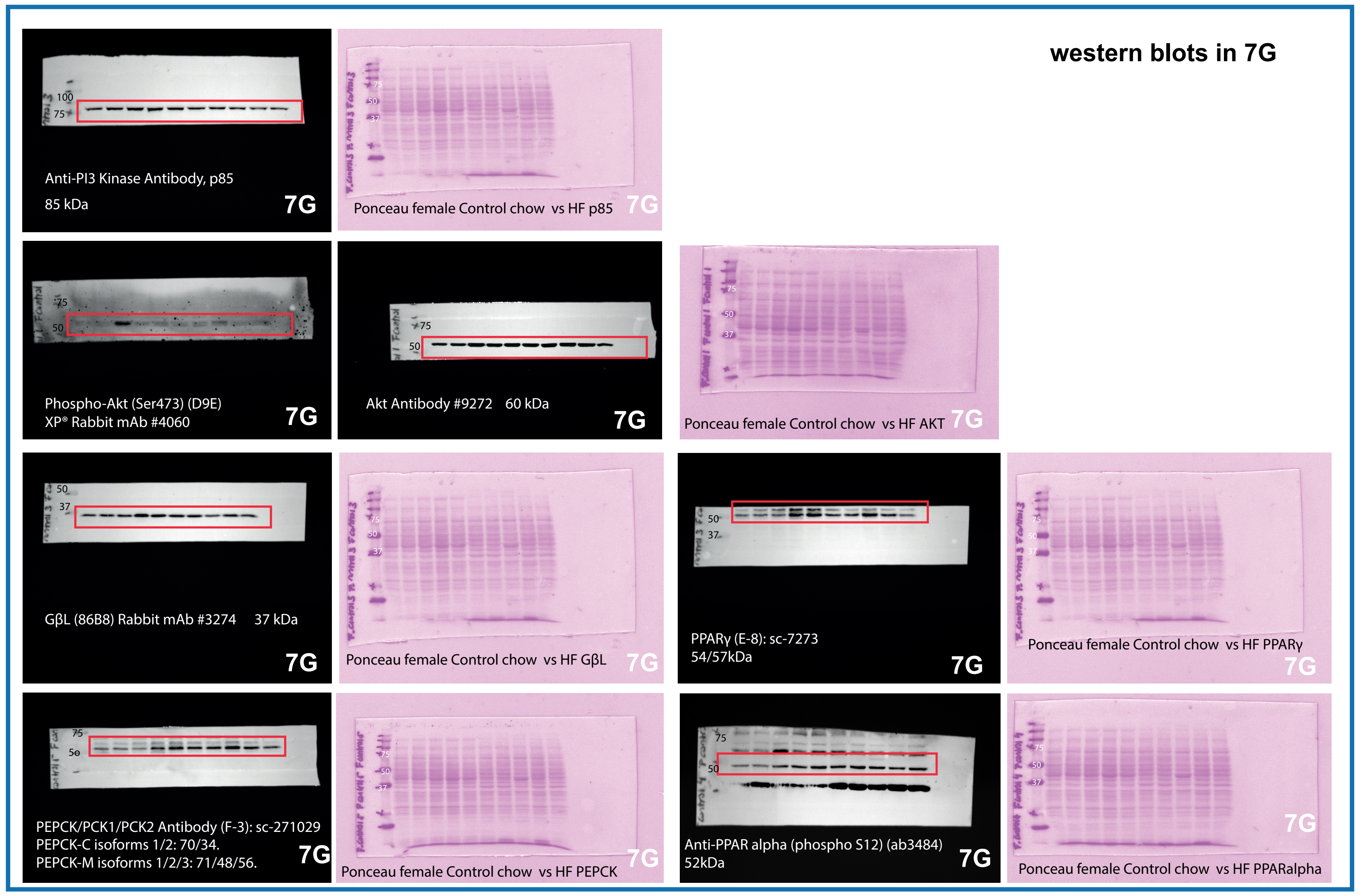
western blots in 7G
Anti-PI3 Kinase Antibody, p85
85 kDa
7G
Ponceau female Control chow vs HF p85
7G
Phospho-Akt (Ser473) (D9E)
XP® Rabbit mAb #4060
7G
Akt Antibody #9272 60 kDa
7G
Ponceau female Control chow vs HF AKT
7G
GβL (86B8) Rabbit mAb #3274 37 kDa
7G
Ponceau female Control chow vs HF GβL
7G
PPARγ (E-8): sc-7273
54/57kDa
7G
Ponceau female Control chow vs HF PPARγ
7G
PEPCK/PCK1/PCK2 Antibody (F-3): sc-271029
PEPCK-C isoforms 1/2: 70/34.
PEPCK-M isoforms 1/2/3: 71/48/56.
7G
Ponceau female Control chow vs HF PEPCK
7G
Anti-PPAR alpha (phospho S12) (ab3484)
52kDa
7G
Ponceau female Control chow vs HF PPARalpha
7G

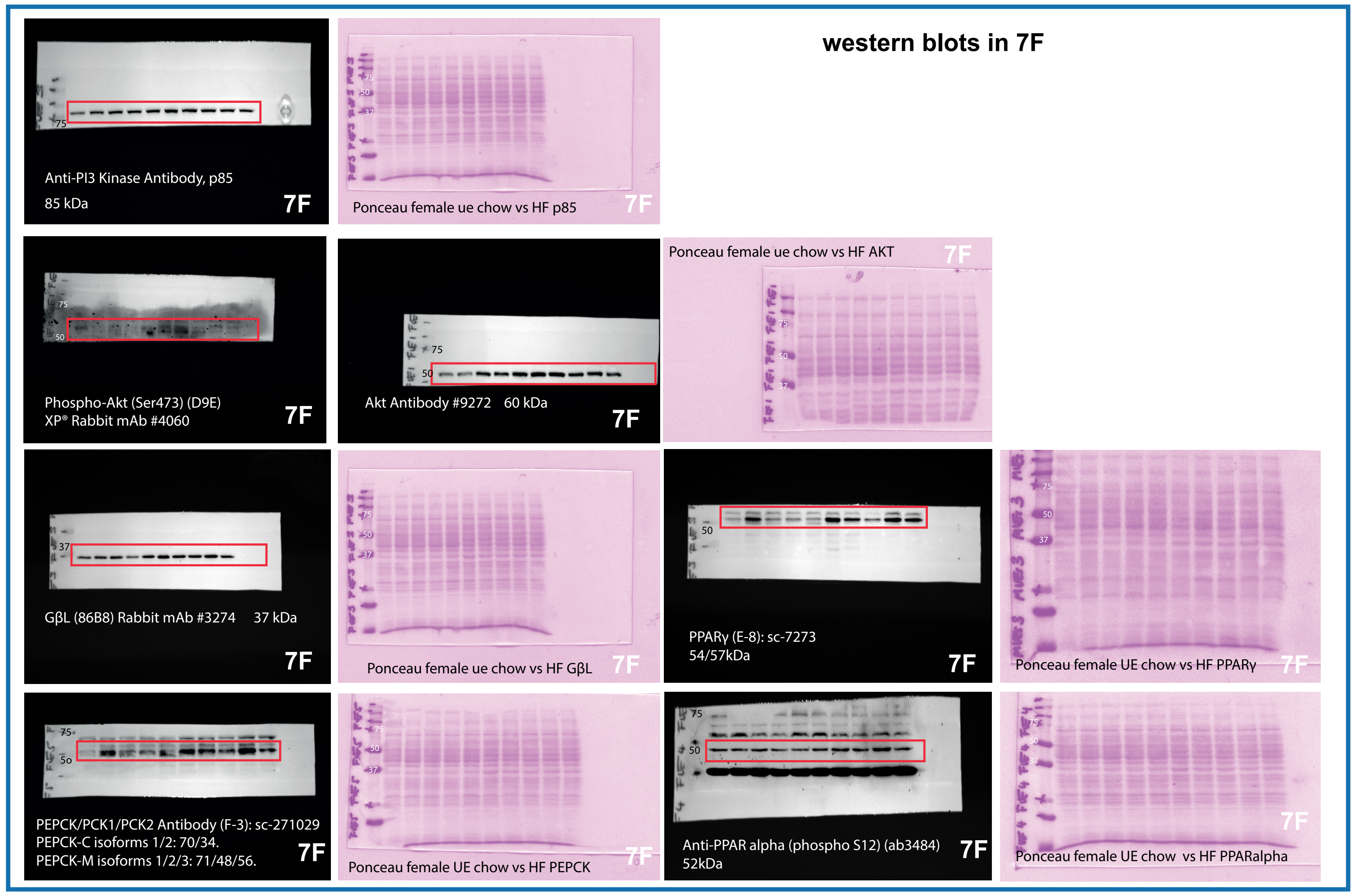
western blots in 7F
Anti-PI3 Kinase Antibody, p85
85 kDa
7F
Ponceau female ue chow vs HF p85
7F
Phospho-Akt (Ser473) (D9E)
XP® Rabbit mAb #4060
7F
Akt Antibody #9272 60 kDa
7F
Ponceau female ue chow vs HF AKT
7F
GβL (86B8) Rabbit mAb #3274 37 kDa
7F
Ponceau female ue chow vs HF GβL
7F
PPARγ (E-8): sc-7273
54/57kDa
7F
Ponceau female UE chow vs HF PPARγ
7F
PEPCK/PCK1/PCK2 Antibody (F-3): sc-271029
PEPCK-C isoforms 1/2: 70/34.
PEPCK-M isoforms 1/2/3: 71/48/56.
7F
Ponceau female UE chow vs HF PEPCK
7F
Anti-PPAR alpha (phospho S12) (ab3484)
52kDa
7F
Ponceau female UE chow vs HF PPARalpha
7F
